# Subclinical endometritis in dairy cattle is associated with distinct mRNA expression patterns in blood and endometrium

**DOI:** 10.1371/journal.pone.0220244

**Published:** 2019-08-02

**Authors:** Mariam Raliou, Doulaye Dembélé, Anna Düvel, Philippe Bolifraud, Julie Aubert, Tristan Mary-Huard, Dominique Rocha, François Piumi, Sophie Mockly, Maike Heppelmann, Isabelle Dieuzy-Labaye, Peter Zieger, David G. E. Smith, Hans-Joachim Schuberth, Iain Martin Sheldon, Olivier Sandra

**Affiliations:** 1 UMR BDR, INRA, ENVA, Université Paris Saclay, Jouy en Josas, France; 2 Institut de Génétique et de Biologie Moléculaire et Cellulaire, CNRS UMR 7104—Inserm U 964—Université de Strasbourg, Illkirch, France; 3 Immunology Unit, University of Veterinary Medicine, Foundation, Hannover, Hannover, Germany; 4 AgroParisTech, UMR 518 MIA, Paris, France; 5 GABI, INRA, AgroParisTech, Université Paris-Saclay, Jouy-en-Josas, France; 6 University of Veterinary Medicine Hannover, Foundation, Clinic for Cattle, Endocrinology Laboratory, Hannover, Germany; 7 Zoetis, Zaventem, Belgium; 8 Zoetis, Berlin, Germany; 9 Institute of Infection, Immunity and Inflammation, College of Medical, Veterinary and Life Sciences, University of Glasgow, Glasgow, United Kingdom; 10 Institute of Life Science, Swansea University Medical School, Swansea University, Swansea, United Kingdom; University of Florida, UNITED STATES

## Abstract

Cattle with subclinical endometritis (SCE) are sub-fertile and diagnosing subclinical uterine disease remains a challenge. The hypothesis for this study was that endometrial inflammation is reflected in mRNA expression patterns of peripheral blood leucocytes. Transcriptome profiles were evaluated in healthy cows and in cows with SCE using circulating white blood cells (WBC) and endometrial biopsy samples collected from the same animals at 45–55 days postpartum. Bioinformatic analyses of microarray-based transcriptional data identified gene profiles associated with distinct biological functions in circulating WBC and endometrium. In circulating WBC, SCE promotes a pro-inflammatory environment, whereas functions related to tissue remodeling are also affected in the endometrium. Nineteen differentially expressed genes associated with SCE were common to both circulating WBC and the endometrium. Among these genes, transcript abundance of immune factors *C3*, *C2*, *LTF*, *PF4* and *TRAPPC13* were up-regulated in SCE cows at 45–55 days postpartum. Moreover, mRNA expression of *C3*, *CXCL8*, *LTF*, *TLR2* and *TRAPPC13* was temporally regulated during the postpartum period in circulating WBC of healthy cows compared with SCE cows. This observation might indicate an advantageous modulation of the immune system in healthy animals. The transcript abundance of these genes represents a potential source of indicators for postpartum uterine health.

## Introduction

In cattle, the postpartum period is associated with uterine tissue remodeling, restoration of immunological homeostasis and resumption of ovarian cyclicity necessary for subsequent fertility [1]. Unfortunately, postpartum infection of the uterus with pathogenic bacteria disrupts these physiological events in many dairy cattle [2], leading to the development of uterine diseases. Whilst the effects of clinical uterine diseases are often self-evident, subclinical uterine disease can also cause sub-fertility [3].

In dairy cattle, multi-pathogen bacterial infections of the genital tract occur after parturition [4]. During bacterial infection the immune cells and endometrial cells generate a local immune response against the pathogens to eliminate them from the uterus [2]. However, nearly half of animals develop some forms of postpartum uterine clinical disease, metritis or clinical endometritis, associated with a perturbed immune function [4,5]. More than 40% of animals also develop subclinical endometritis (SCE) [6] defined as an inflammation of the uterine endometrium, that can be detected by histology or cytology, in the absence of purulent material in the vagina [4].

The endometrium is not only a biological sensor able to fine-tune its physiology in response to the presence of embryo [7,8], but it represents the first line of defence against invading bacteria. Bovine epithelial and stromal cells of endometrial origin expressed Toll-like receptors (TLRs) suggesting that these cells have the potential to recognize and respond to a bacterial infection via these receptors [9,10,11,12]. The TLRs and their associated inflammatory mediators are known to contribute to endometritis and pregnancy disorders [13]. In addition, endometritis has also been associated with altered levels of pro-inflammatory cytokines gene expression due to impaired activation of inflammation and clearance of uterine bacteria [14]. Microarray studies have also reported that clinical endometritis and SCE were associated with modified expression levels of endometrial genes linked to the immune system, cell adhesion, regulation of apoptotic signaling, G-protein coupled receptors signaling pathways and chemotaxis in cattle [15]. Interleukins (*IL6*, *IL1*, and *TNFA)* and chemokines (*CXCL5* and *CXCL8)* have been suggested as potential endometrial biomarkers for SCE in cattle [16]. It has been reported that the immune capability of the uterus is influenced by steroid hormones, especially luteal progesterone, which increases the susceptibility of the postpartum endometrium to infection in dairy cattle and sheep [17,18,19]. Nevertheless, robustness of endometrial biomarkers for SCE when considering reproductive cycle has not been investigated.

Subclinical endometritis may not only be associated with modifications of molecular pathways in the endometrium but also in the systemic environment. Postpartum SCE at 45–55 days post-partum was associated with an increase in total number of blood mononuclear cells and the higher expression of genes encoding inflammatory mediators, including *TNF*, *IL12* and *CXCL8* [20], complement proteins (*C2*, *C3*), pathogen recognition receptors (*TLR2*, *TLR4*), antimicrobial peptides (*DEFB*, *S100A8*) and acute phase proteins (*SAA1*, *SAA3*) [5]. Conversely, lower levels of peripheral blood cell expression of *IL1B* may contribute to the development of metritis [21]. In addition, a recent study demonstrated that the levels of TNF-α, IL-6, IL-10, as well as acute phase proteins (Hp) and serum amyloid A (SAA), were significantly higher in the serum of cows with SCE compared with control animals in early post-partum [22]. However, it remains unclear how changes in gene profile of the endometrium relates to circulating white blood cells in animals with SCE [23].

The hypothesis for the present study was that endometrial inflammation was reflected in peripheral blood leucocytes at the transcriptional level. We aimed to determine and to explore the mRNA expression patterns in circulating white blood cells and endometrial biopsy samples collected from the same animals at 45–55 days postpartum (DPP). Using a microarray approach, global mRNA expression profiling was performed to identify genes and biological functions with altered expression in endometrium and in circulating white blood cells (WBC) from Holstein cows with postpartum SCE.

## Material and methods

### Ethics statement

This study was approved by the Niedersächsisches Landesamt für Verbraucherschutz und Lebensmittelsicherheit (AZ 33.9-42502-05-09A/598). All procedures involving animals were carried out in accordance with German legislation on animal welfare.

### Study design, groups of animals, and source of bovine endometrium tissue

The study involved 26 multiparous Holstein-Friesian cows, between 4 and 10 years old, housed at the Farm for Education and Research at the University of Veterinary Medicine Animals (Hannover, Germany), as described [20]. No cow with clinical signs of pathology including post-parturient metritis, retained fetal membranes or mastitis was included in the study. All cows were followed from calving (Day 0) to 55 days postpartum (DPP). For blood sampling and collection of endometrial tissue, a complete general examination was carried out from 45 to 55 DPP. The gynecologic examination was performed at the time of sampling. To identify the subclinical animals (SCE) both histological and cytobrush analyses were performed as described [20]. Histological samples from cows were analyzed for the presence and semi-quantitative abundance of neutrophilic and eosinophilic granulocytes, lymphocytes, macrophages, and plasma cells. Based on the histology-based diagnosis of non-purulent endometritis at 45 to 55 DPP and in the absence of clinical signs of endometritis, cows were classified as “subclinical endometritis” (SCE, n = 9) or healthy (n = 17). For 22 animals (healthy, n = 14, SCE, n = 8), composition in immune cell types was determined in the circulating white blood cells fraction and proportion of the major cell types in the endometrial biopsies was determined based on their morphological appearance as previously described [20].

### Collection of endometrial biopsy samples

The stage of the estrous cycle was specified by ultrasonography as described previously [20]. Among 26 examined cows, the stage of estrous cycle could not be successfully specified for 8 of them and they were classified as “undetermined”. Whether a corpus luteum was detectable or absent, the remaining cows (n = 18) were considered to be in the luteal phase (LP; healthy, n = 6; SCE, n = 4) or in the follicular phase (FP; healthy, n = 7; SCE, n = 1) (S1 Table). At 45–55 DPP, endometrial biopsies were collected transcervically from the base of the uterine horn ipsilateral to the corpus luteum for the 5 SCE cows and the 10 healthy cows for which the stage of estrous cycle was unambiguously determined. Endometrial biopsies were immediately transferred into nitrogen (-169°C) then subsequently stored at -80°C for molecular analyses.

### Blood sampling using PAXgene

Blood was collected from the jugular vein into PAXgene Blood RNA Tubes (Becton Dickinson, Heidelberg, Germany) using a Becton Dickinson Vacutainer System (Becton Dickinson, Heidelberg, Germany) according to the manufacturer’s instructions. Among the 26 females enrolled in this study, analysis of immune cell types in blood was carried out for 22 cows (healthy, n = 14; SCE, n = 8) at 45–55 days post-partum (DPP, S1 Fig). At 45–55 days post-partum, for molecular analyses, total RNA material was available from 17 females (healthy, n = 10; SCE, n = 7) and from 15 females (endometrial biopsies). Regarding the time-course of RNA expression in peripheral blood cells, total RNA material was available at the three time points (1–4, 9–11 and 45–55 DPP) using PAXgene blood samples collected from 14 females (healthy, n = 10; SCE, n = 4; S1 Table). Immediately after blood collection, the PAXgene Blood RNA tubes were inverted gently eight to 10 times, stored between 2 and 6 hours at room temperature and frozen at -20°C for 24 hours followed by storage at -80°C as described previously [20].

### RNA isolation

Total ribonucleic acid (RNA) was isolated from endometrial biopsies as described previously [20]. Total RNA was isolated from PAXgene blood samples using the PAXgene Blood RNA system (PreAnalytiX, Qiagen/BD Company, France) according to the manufacturer’s instructions (manual procedure). Each PAXgene tube was incubated at room temperature for 2 hours and then centrifuged for 10 minutes at 4000 × *g* to remove red blood cells in the supernatant. The pellet was washed twice, resuspended in lysis buffer and incubated with protease K solution for 10 minutes to remove proteins. Samples were centrifuged for 3 minutes at 12000 × g and washed with 70% ethanol. Isolated total RNA was treated with 1 μl of DNase (RNase-Free DNase Set; Qiagen) for 15 minutes at room temperature to remove genomic DNA contamination. Quality and integrity of the total isolated and purified RNA were assessed using an Agilent 2100 Bioanalyzer (@bridge ICE -Iso Cell Express-, INRA, France: https://www6.jouy.inra.fr/ice). The RNA integrity number (RIN) of each RNA sample ranged from 8.5 to 9.5.

### Custom bovine gene expression microarray processing and hybridization

Transcriptional profiling was performed using a custom bovine array generated by merging the Agilent bovine 44K microarray (National Center for Biotechnology Information, Gene Expression Omnibus accession no 023647_B. taurus Oligo Microarray V2_1 and V2_4) with a non-commercial bovine INRA 22K (13,257-element bovine oligonucleotides array, Gene Expression Omnibus accession no. GPL2853). This custom bovine microarray was designed based on bovine annotated Ensembl transcripts (http://www.ensembl.org/index.html, genome assembly UMD3.1) and represented 19,479 transcripts. It was manufactured by Agilent Technologies using 60-mer oligonucleotides probes (Agilent eArray software; https://earray.chem.agilent.com/earray/) and an *in situ* synthesis printing process.

RNA labeling was performed using total RNA samples isolated from endometrial biopsies and WBC collected from the same animals, namely 4 SCE cows (3 females in LP; 1 female in FP) and 4 healthy cows (2 females in LP; 2 females in FP). In the SCE group, four females (FP, n = 1; LP, n = 3) were randomly drawn. In the “healthy” group, two females were randomly drawn in the “luteal phase” sub-group and two were randomly drawn in the “follicular phase” sub-group. The Agilent protocol “One-Color Microarray-Based Gene Expression Analysis–Low Input Quick Amp Labelling” version 6.5, May 2010 (Cat # G4140-90050) was used for RNA labeling. Briefly, complementary RNA (cRNA) samples were linearly amplified and labelled with cyanine 3 starting from 100 ng of total RNA. Following fragmentation, labelled cRNA were hybridized on the custom bovine gene expression array (8x60K) for 17 hours, rotating at 10 rpm for 65°C. Following washing, slides were scanned using an Agilent G2565CA microarray Scanner System, at a 3 μm resolution in a 20-bit scan mode, according to the “AgilentG3_GX_1Color” protocol. The microarray data were submitted to GEO (Gene Expression Omnibus) database (accession number GSE115667).

### Microarray analysis

Raw.tiff images were extracted using Agilent “Feature Extraction, version 10.10.1.1” following the “GE1_1010_Sep10” protocol. Median raw expression values were further normalized by the quantile method [24,25]. Differentially expressed genes (DEGs) were selected using the fold change rank ordering (FCROS) method [26] and the t-test relative to a threshold–TREAT- option of the LIMMA method [27]. The p-values obtained using TREAT were adjusted using the Benjamini & Hochberg method [28]. Finally a threshold is used to select significant genes.

### Principal component analyses and hierarchical clustering

Principal Component Analyses (PCA) were performed using the FactoMineR package [29] to check the dispersion estimates of the individual samples using the log2 transformed probe values. For each PCA, confidence ellipses around the categories are for the means of the categories (the empirical variance is divided by the number of observations) with a confidence level of 0.95.

### Ingenuity pathway analysis

Pathway analyses were performed to identify associations of the genes identified in WBC and endometrium studies with canonical pathways and gene networks. The pathways and networks were generated with Ingenuity Pathway Analysis (IPA; Ingenuity Systems, www.ingenuity.com). Two lists of genes, which were differentially expressed between SCE and healthy animals in WBC and endometrium at 45–55 DPP, were uploaded to the IPA tool. The IPA functional analysis tools identified the biological functions and/or pathways that were most significant (*p-value* < 0.05) according to a right-tailed Fisher exact test. The analysis includes canonical pathways overrepresented in gene lists, the integration of pathways into biological networks; predicted upstream regulators implicated in the cascade of mRNA expression changes. A *p-value* for a given function was calculated by considering the number of functional analysis molecules that participate in that function and the total number of molecules that are known to be associated with that function/pathway in the Ingenuity Knowledge Base. Functions and pathways with *p-value* < 0.05 were considered significant for a given list of differentially expressed genes (DEGs). The ratio indicates the number of genes for a particular pathway/ontology in a gene list compared to the total number of genes in a given pathway. Interaction networks for each list of DEGs were generated by identifying genes that serve as molecules of interest. These molecules of interest interact with other molecules in the Ingenuity Knowledge Base, and were identified as network-eligible molecules and used as molecules to generate a network.

### Analysis of mRNA expression by quantitative real-time PCR

Expression levels of selected candidate DEGs in circulating WBC (n = 9 DEGs) and in endometrial biopsy samples (n = 12 DEGs) were analyzed in SCE and healthy cows at 45–55 DPP. Quantitative real time PCR (RT-qPCR) was performed using circulating WBC (SCE, n = 7; healthy, n = 10) and available endometrial biopsies (SCE, n = 3; healthy, n = 10). For the RT-qPCR analyses carried out with WBC sampled from SCE cows, the 7 females were classified as “luteal” (n = 4), “follicular” (n = 1) or undetermined (n = 2). For the endometrial samples, the 3 SCE females were classified as “luteal”. For healthy animals, endometrial and WBC samples were collected from females in follicular phase (n = 5) and from females in luteal phase (n = 5) (S1 Table).

Temporal regulation of the selected candidate DEGs (n = 9) was investigated in circulating WBC collected at 1–4, 9–11 and 45–55 DPP of 10 healthy females (LP, n = 3, FP, n = 6 and undetermined, n = 1) and 4 SCE females (LP, n = 1; FP, n = 1; undetermined, n = 2). Specific primers (S2 Table) were designed for every candidate gene using NCBI Primer-BLAST [30]. To assess that cDNA amplification generated the expected fragment, every amplicon was sequenced and blasted on NCBI RNA bovine database to confirm its identity. First-strand complementary DNA (cDNA) was synthesized from 500 ng of purified total RNA using 0.025 μg Oligo(dT)_12-18_ as primers and 10U of SuperScript II Reverse Transcriptase enzyme (Invitrogen, France) in a 20 μl reaction volume according to the manufacturer’s instructions. The PCR reactions, consisting of 50 ng of cDNA, 15 μM concentration of primers and 7.5 μl of SYBR green Mastermix (Applied Biosystems), were carried out using the StepOnePlus Real-Time PCR System (Applied Biosystems, France), according to the relative standard curve method [31]. All PCR reactions were carried out in duplicate with a final reaction volume of 15 μl made up with RNase DNase-free water under the following cycling conditions: denaturation at 95°C for 10 minutes, followed by 45 cycles consisting of denaturation at 95°C for 15 seconds, annealing at 60°C for 60 seconds and DNA synthesis at 72°C for 40 seconds. Specificity for reaction products was controlled with their respective melting curves. A standard curve was included for each gene to generate arbitrary expression values for candidate genes. The Qbase software (Biogazelle,) based on the geNorm module [32] was used for analyzing quantitative PCR data. The geNorm module determined the expression stability of candidate references and the optimal number of reference genes. Five genes (*ACTB*, *B2M*, *GAPDH*, *RLP19* and *TPRG1L*) were tested to identify the most stable genes under the experimental conditions used in this study. Glyceraldehyde-3-phosphate dehydrogenase (*GAPDH*) and actin beta (*ACTB*) were the most stable reference genes in WBC and *GAPDH* and tumor protein p63 regulated 1 like *(TPRG1L*) in endometrium. All expression data for gene transcripts of interest were expressed as mean calibrated normalized relative quantity (CNRQ) values in arbitrary units.

### Statistical analyses

Statistical analyses were performed using Prism software version 6.07 GraphPad. Normal distribution of data was tested using D'Agostino & Pearson test and Shapiro-Wilk test. Normalized and calibrated values of mRNA levels were analyzed with a Mann-Whitney U test to compare expression abundance of candidate genes between SCE and healthy cows. Array analysis indicated a specific prediction about the direction of the genes expression between SCE and healthy cows, so one-tailed test was performed on the data. Two-way Repeated Measures (RM) ANOVA matched values, Sidak’s multiple comparisons test was used to compare paired samples within the group at 1–4, 9–11 and 45–55 DPP. Values of P < 0.05 were considered to be statistically significant. The transcript level of candidate genes was represented as bars using Prism software version 6.07 GraphPad.

## Results

### Composition of peripheral whole blood cells

At 45–55 DPP, cell type composition of circulating WBC was determined for the 22 Holstein dairy cows enrolled in the study. Cows with subclinical endometritis (SCE, n = 8) showed a significant higher number of leucocytes, lymphoids cells and monocytes (S1 Fig) compared with healthy cows (n = 14). In healthy cows for which the stage of estrous cycle was defined and distribution of immune cells was available (n = 10), the number of peripheral blood cells for the major categories was similar between cows in luteal phase (n = 4) and cows in follicular phase (n = 6), (S1 Fig). Based on the number of main categories of circulating blood cells (leucocytes, polymorphonuclear cells, lymphoid cells and monocytes), the principal component analysis (PCA) showed a more dispersed distribution for the SCE cows than for the healthy cows (S1 Fig).

### Circulating white blood cells and endometrium exhibit specific transcriptome profiling in dairy cattle

**Fig 1:**
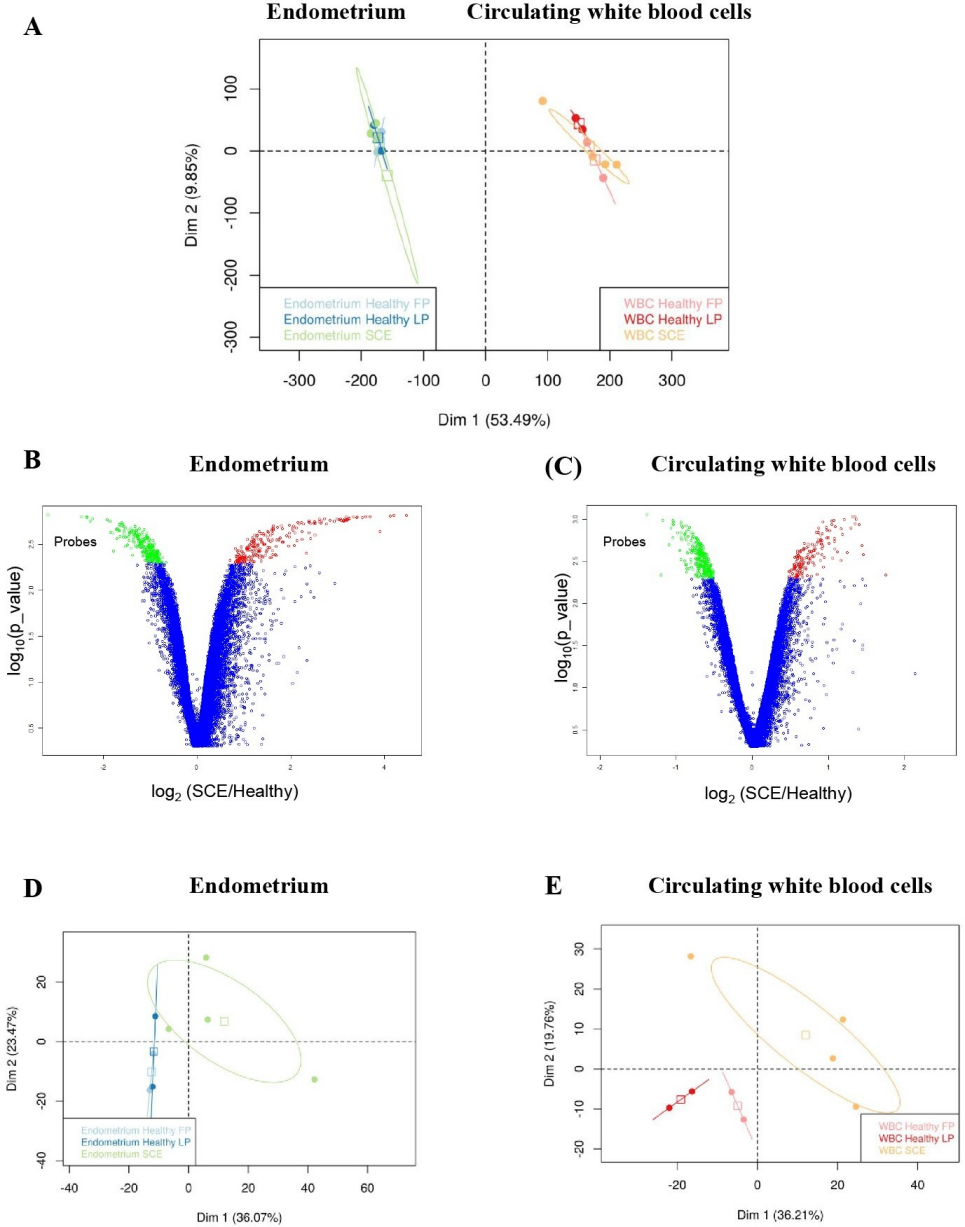
Gene expression profiles for endometrium and circulating white blood cells collected from Holstein dairy cows with subclinical endometritis and from healthy cows. (**A**) Principal component analysis (PCA) plots using all genes (probes) from microarray-based data obtained from endometrium and circulating white blood cells (WBC) collected from the same individuals (dairy Holstein cows) at 45–55 days postpartum (DPP). Plots and cluster distributions on 19,479 gene patterns are presented for endometrial biopsies and circulating white blood cells collected from 4 cows with subclinical endometritis (SCE; follicular phase, n = 1; luteal phase, n = 3) and from 4 healthy cows either in follicular phase (FP; n = 2) or in luteal phase (LP; n = 2). All SCE females were gathered in a unique group. The PCA score plots showed segregation between endometrium and WBC. Endometrial samples from SCE cows, healthy LP cows and healthy FP cows are indicated in green, dark blue and light blue color respectively. Circulating white blood cells (WBC) from SCE cows, healthy LP cows and healthy FP cows are indicated in orange, red and pink color respectively. (**B, C)** Volcano plot analyses were used to show the differentially expressed genes in endometrium and in circulating white blood cells from cows with subclinical endometritis (SCE, n = 4) compared with healthy (n = 4) Holstein dairy cows at 45–55 days postpartum (DPP) respectively. The x-axis represents the log2 of the fold change (FC), which was plotted against the −log10 of the p-value. The p-value is equal to the f.value for down regulated genes or 1-f.value for up-regulated genes. The f.value thresholds 0,005 and 0,995 were used for down- or up-regulated genes (in green and red respectively). These threshold values lead to a selection error equal to 1%. (**D, E**) Principal component analysis (PCA) plot using differentially expressed genes (DEGs) identified in endometrium and circulating white blood cells from 4 Holstein dairy cows with subclinical endometritis (SCE) compared with 4 healthy Holstein dairy cows in luteal phase (LP; n = 2) or in follicular phase (FP; n = 2) at 45–55 days postpartum (DPP). (**D**) Endometrial biopsies from SCE cows (n = 4), healthy LP cows (n = 2) and healthy FP cows (n = 2) are indicated in green, dark blue and light blue color respectively. (**E**) Circulating white blood cells (WBC) from SCE cows (n = 4), healthy LP cows (n = 2) and healthy FP cows (n = 2) are indicated in orange, red and pink color respectively.

Using a custom bovine gene expression array containing 19,479 transcripts, gene expression patterns were determined in circulating white blood cells (WBC) and endometrium of Holstein dairy cows with subclinical endometritis (SCE, n = 4) and healthy Holstein dairy cows (n = 4) at 45–55 days post-partum. WBC and endometrial biopsies were analyzed for each of the eight females. Principal component analyses (PCA) performed with all genes (probes) revealed a clear discrimination between endometrium and WBC transcriptomic profiles in both SCE and healthy animals (Fig 1A). Based on these analyses, transcriptomic profiles between SCE cows and healthy cows were not distinguishable in the endometrium nor in circulating WBC.

### Subclinical endometritis is associated with specific mRNA expression patterns for circulating white blood cells and endometrium of dairy cattle

Volcano plots that combined fold change (FC) and probabilities (f-value *<* 0.005 for down regulated probes and f-value > 0.995 for up-regulated probes) from the fold change rank ordering statistics (FCROS) method (FDR = 1%) were used to visualize the distribution of differentially expressed genes (DEG) between SCE and healthy cows in circulating WBC and endometrium collected at 45–55 DPP. The down-regulated and up-regulated probes were indicated in green and red color respectively (Fig 1B and 1C). By using a method based on a linear model (t-test relative to a threshold-TREAT- p-value ≤ 0.05, FDR = 5%) [27] the comparison of expression profiles between SCE cows and healthy cows identified 512 DEGs in endometrium (S3 Table) and 340 DEG in circulating white blood cells (S4 Table). Considering the 512 DEGs identified in the endometrium, 281 and 231 DEG displayed a higher expression level in the healthy cows group and in the SCE cows group respectively (FC > +1.2, p-value ≤ 0.05). For the 340 DEG in the circulating WBC, 171 and 169 DEGs displayed a higher transcript expression in the healthy cows group and in the SCE cows group respectively (FC > +1.2, p-value ≤ 0.05). Principal component analysis (Fig 1D and 1E) run with DEGs in circulating WBC showed a more clear separation between SCE cows and healthy cows compared with PCA run with endometrial DEG, as confirmed by the hierarchical clustering (data not shown). Healthy cows sampled during the luteal phase (LP) appeared closer to healthy females sampled during the follicular phase (FP) in the endometrium than in the WBC.

**Fig 2:**
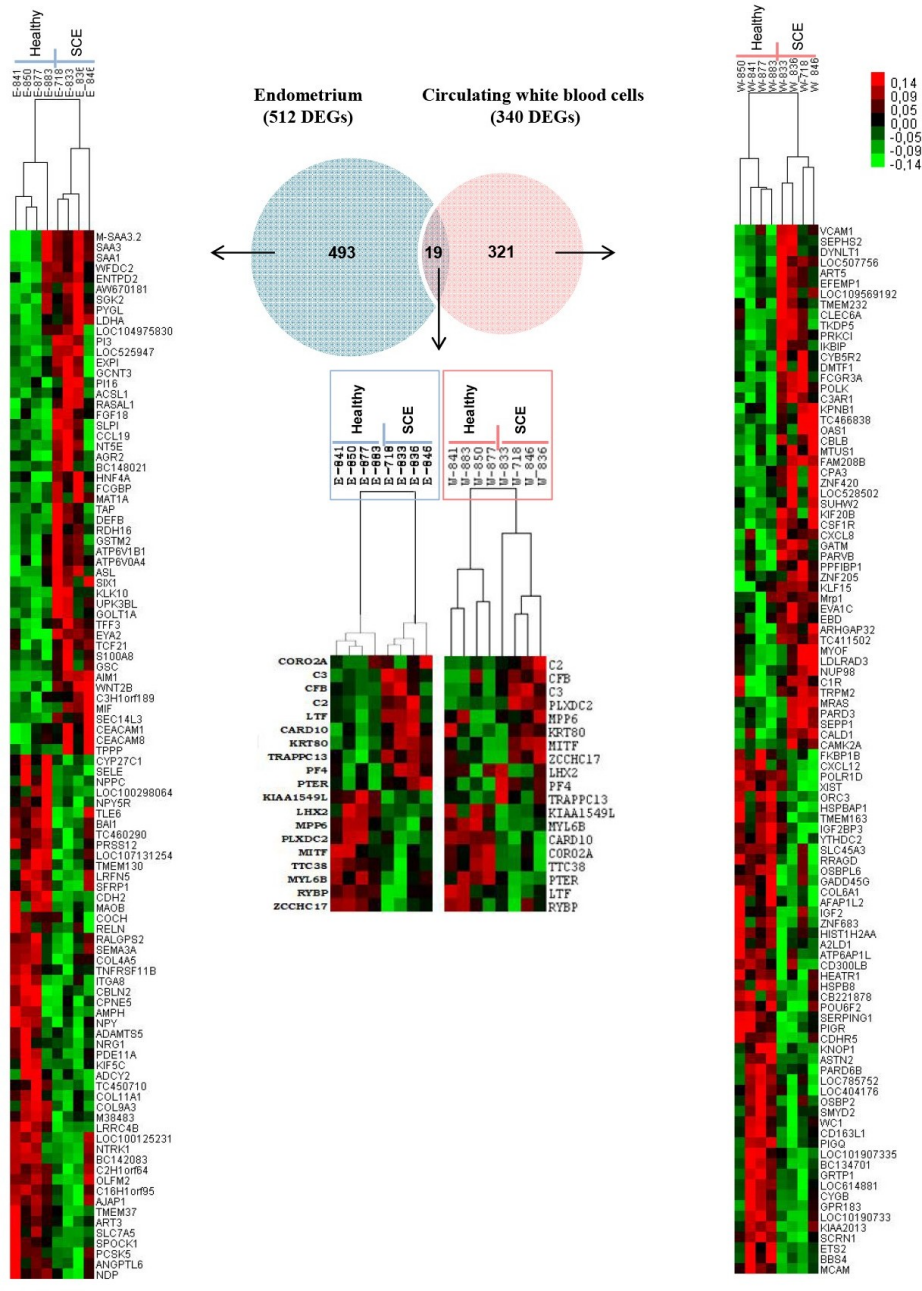
Venn diagram and Heat maps showing the molecular effects of subclinical endometritis on mRNA expression in endometrium and in circulating white blood cells (WBC). Differentially expressed genes between Holstein dairy cows with subclinical endometritis (SCE, n = 4 including follicular phase, n = 1; luteal phase, n = 3) and healthy (n = 4 including follicular phase, n = 2; luteal phase, n = 2) Holstein dairy cows were identified at 45–55 days postpartum (DPP) in endometrium and the WBC. In the Venn diagram, the number of DEG is indicated in brackets. For the endometrium and the WBC, the top 100 DEG (fold change ≥ ±1.5, t-test relative to a threshold probability, p-value ≤ 0.05) are presented as heat maps (Cluster and TreeView software). Heat map expression values are represented as colors, where red squares indicate up-regulated DEGs and green squares indicate down-regulated DEGs.

Based on the DEGs identified between SCE and healthy cows at 45–55 DPP, a Venn diagram (Fig 2) indicated that 493 DEGs, 321 DEGs and 19 DEGs (FC ≥ ±1.2; p-value ≤ 0.05) were endometrium-specific, circulating WBC-specific and common between WBC and endometrium respectively (S3 and S4 Tables). Heat maps (Fig 2) illustrated an overview of the top 100 DEGs (50 up- and 50 down-regulated DEGs) found in the endometrium (left panel) and circulating WBC (right panel). Among the 19 DEGs common between endometrium and circulating WBC, 11 DEG displayed a regulation that was similar in WBC and endometrium of SCE animals (6 up-regulated DEG: *C2*, *C3*, *CFB*, *KRT80*, *PF4*, *TRAPPC13*; 5 down-regulated DEG: *LHX2*, *KIAA1549L*, *MYL6B*, *RYBP*, *TTC38*) whereas 8 DEG (*CARD10*, *CORO2A*, *LTF*, *MITF*, *MPP6*, *PLXDC2*, *PTER*, *ZCCHC17)* exhibited an opposite regulation (Fig 2).

#### Gene networks associated with mRNA expression changes in the endometrium of cows with subclinical endometritis

The Networks module of IPA was used to determine networks associated with DEG identified in the endometrium. Twenty five networks (S5 Table) were identified in the endometrium (p-value ≤ 0.05). Three major networks related “Amino Acid Metabolism, Small Molecule Biochemistry, Lipid Metabolism”, Cell-To-Cell Signaling and Interaction, Cellular Movement, Hematological System Development and Function” and “Digestive System Development and Function, Organismal Functions, Lipid Metabolism” are presented in S2 Fig.

#### Biological processes affected in the endometrium of cows with subclinical endometritis

**Fig 3:**
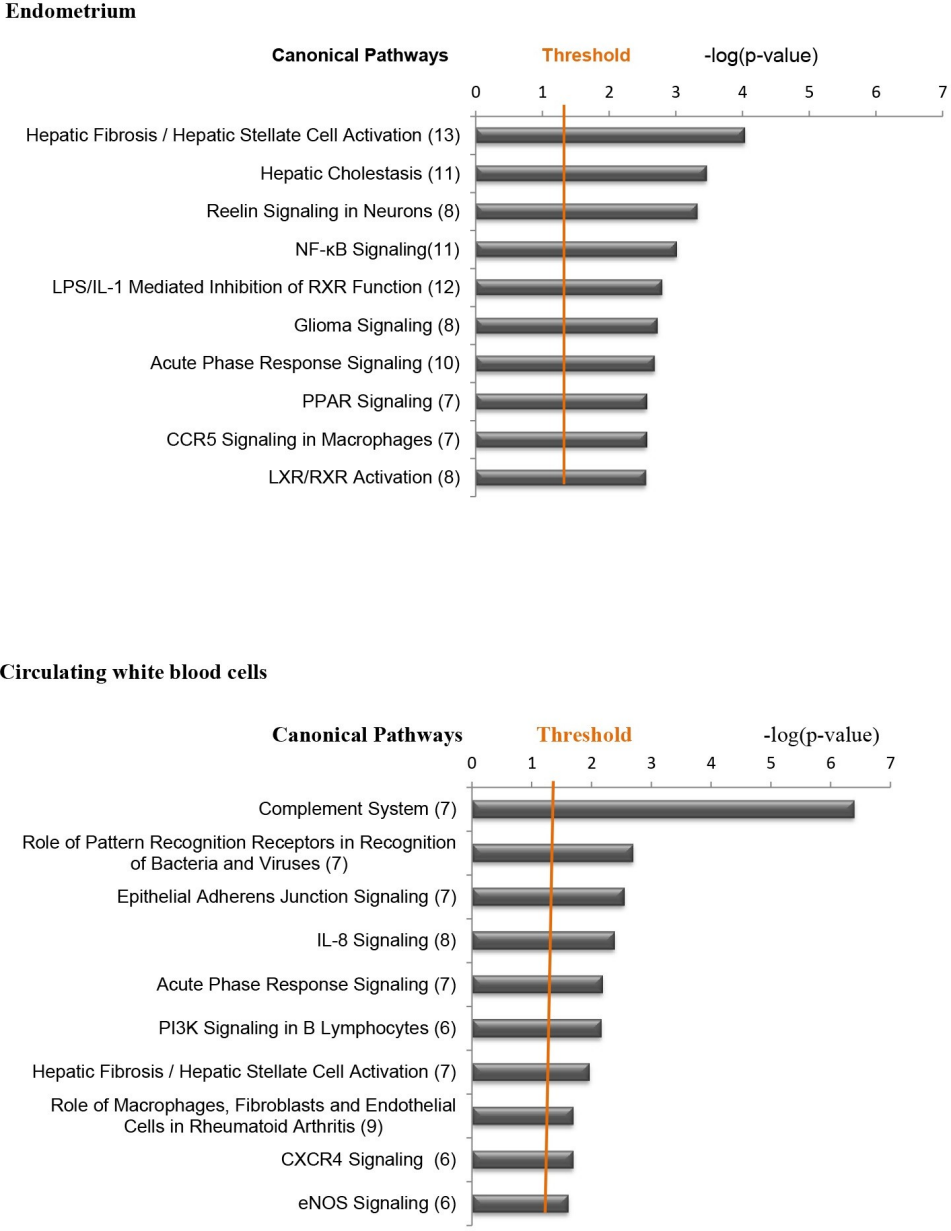
Canonical pathways analysis of differentially expressed genes in cows with subclinical endometritis compared with healthy dairy Holstein cows at 45–55 days postpartum. Ingenuity Pathway Analysis (IPA) was run to determin**e** canonical pathways altered in endometrial biopsies and in white circulating blood cells of cows with subclinical endometritis (SCE, n = 4 including follicular phase, n = 1; luteal phase, n = 3) compared with healthy cows (n = 4 including follicular phase, n = 2; luteal phase, n = 2) at 45–55 days postpartum (DPP). The top-10 canonical pathways are presented with the number of molecules indicated in brackets. *P*s were significant (p < 0.05) for random datasets calculated using Fisher’s exact test. Threshold criteria considered for the analysis are -log(p-value)  > 1.3. The list of genes under each category is provided in S2 Table.

To gain insights into the biological processes altered in the endometrium of SCE cows, the 512 DEG were analyzed using the IPA software (S3 Table). Significant association (p-value ≤ 2.0E-3) was established for 140 “disease and biological function” terms containing n ≥ 30 genes (S6 Table). The five top functions for “diseases and disorders”, “molecular and cellular functions” and “Physiological System Development and Function” categories are presented in table in S7 Table. In cows with SCE compared with healthy cows molecular and cellular functions including “Cell movement of myeloid cells” and “Chemotaxis of myeloid cells” were significantly increased while biological functions related to viral infection and cell-cell contact were significantly decreased (S3 Fig). Fifty three canonical pathways (n ≥ 4 genes, -log(p-value ≥ 1.3; S8 Table) were significantly affected in cows with SCE. The top 10 canonical pathways (Fig 3) include “Hepatic Fibrosis / Hepatic Stellate Cell Activation”, “NF-κB Signaling”, “LPS/IL-1 Mediated Inhibition of RXR Function”, “Acute Phase Response Signaling” and “PPAR signaling”.

#### Identification of upstream regulators linked with mRNA expression changes in the endometrium of cows with subclinical endometritis

**Fig 4:**
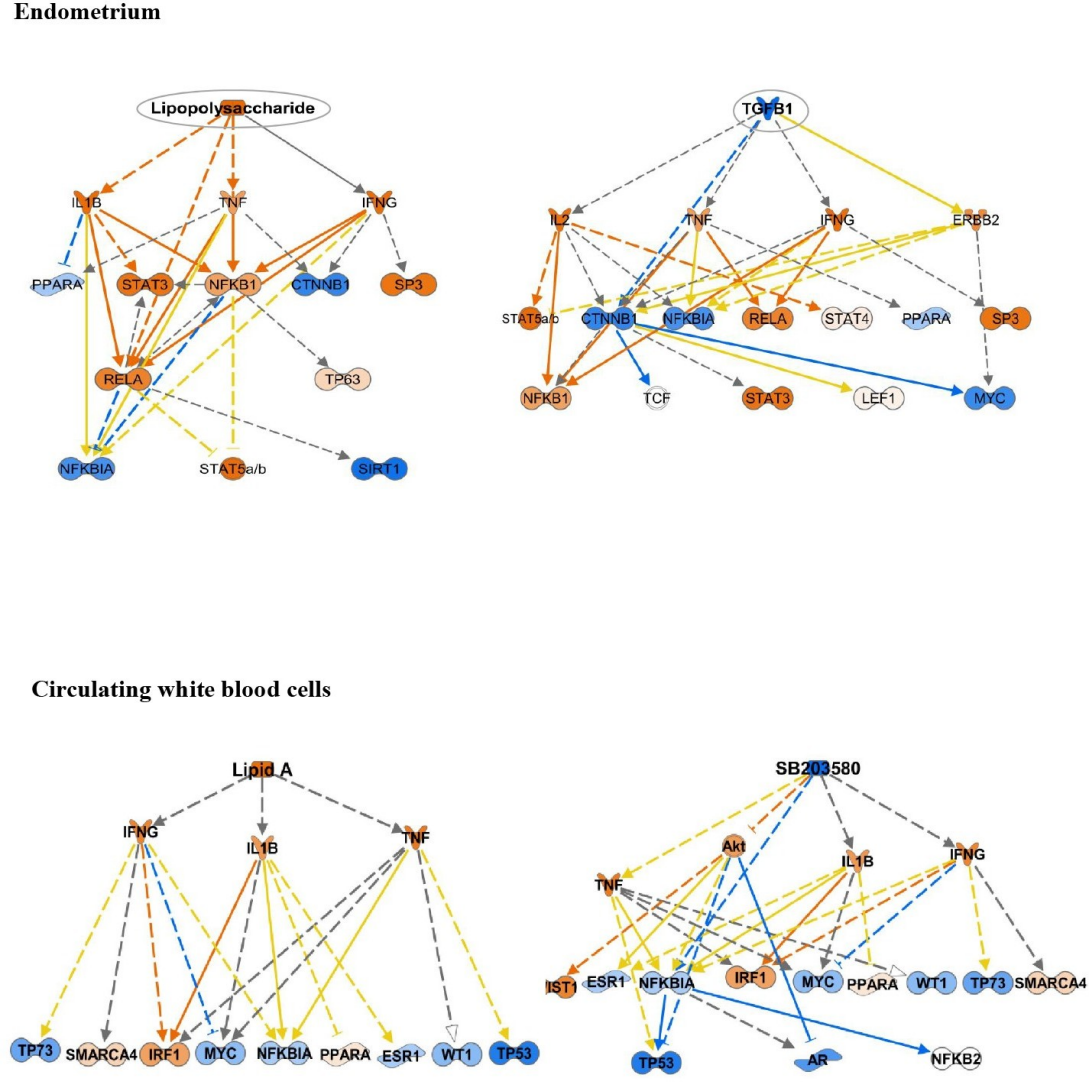
The mechanistic network of the upstream regulators and their relationship predicted by Ingenuity Pathway analysis (IPA). The molecules shown in orange are predicted to be activated (Z-Score ≥ 2, p-value of overlap ≤ 0.05) while the molecules shown in blue are predicted to be inhibited (Z-Score ≥ -2, p-value of overlap ≤ 0.05). Orange and blue dashed lines with arrows indicate indirect activation and inhibition, respectively. Yellow and grey dashed lines with arrows indicate inconsistent effects and no prediction, respectively. (**A**) Lipopolysaccharide (Z-score = +3.37, p-value of overlap = 0.0000894) and (**C**) Lipid A (Z-score = +2.24, p-value of overlap = 0.000268) are predicted to be activated in the endometrium and in the circulating white blood cells of cows with subclinical endometritis (SCE) compared with healthy dairy Holstein cows at 45–55 days postpartum (DPP), respectively. (**B)** TGFB1 (Z-score = -2.55, p-value of overlap = 0.00152) and (**D**) a chemical—kinase inhibitor such SB203580 (Z-score = -2.22, p-value of overlap = 0.00103) are predicted to be inhibited in the endometrium and in the circulating white blood cells of SCE cows compared with healthy cows at 45–55 DPP, respectively. Orange and blue solid lines with arrows indicate direct activation and inhibition, respectively.

The upstream analysis module of IPA identified 133 putative upstream regulators (p-value < 0.05) including cytokines, transcription factors, growth factors, endotoxins and miRNA (S9 Table). Twelve of them were predicted to be significantly associated with a mechanistic network (Z-Score ≥ +2, or Z-Score ≤ -2, p-value of overlap < 0.05; S9 Table). Fig 4 presents two mechanistic networks driven by two putative regulators, namely lipopolysaccharide (Z-score = -2.55, p-value of overlap = 1.52E-05) and growth factor TGFB1 (Z-score = + 3.373, p-value of overlap = 8.94E-03).

#### Gene networks associated with mRNA expression changes in circulating white blood cells of cows with subclinical endometritis

The Networks module of IPA identified 25 networks with the DEGs identified in circulating white blood cells of SCE compared with healthy cows at 45–55 DPP (S10 Table). Three major networks related to “Endocrine System Development and Function, Lipid Metabolism, Small Molecule Biochemistry”, “Humoral Immune Response, Protein Synthesis, Hematological System Development and Function ” and “Cell Cycle, Reproductive System Development and Function, Cardiovascular Disease” are presented in S4 Fig.

#### Biological processes affected in the circulating white blood cells of cows with subclinical endometritis

At 45–55 DPC, the 340 DEG found in the circulating WBC of SCE cows compared with healthy cows were analyzed using the IPA software. Significant association (p-value ≤ 4.0E-3) was established for 83 IPA Disease and biological functions terms with n ≥ 30 genes (S7 and S11 Tables). The five top functions for “diseases and disorders”, “molecular and cellular functions” and “Physiological System Development and Function” categories are presented in table in S7 Table. Biological functions including “Migration of cells” as well as various immune cells-related functions (e. g. cellular infiltration of leucocytes, cell movement of phagocytes and of leucocytes), were significantly increased (S3 Fig). Twenty five canonical pathways (n ≥ 4 genes, -log(p-value) ≥ 1.3) were determined in cows with SCE, including “Complement System”, “Role of Pattern Recognition Receptors in Recognition of Bacteria and Viruses”, “Epithelial Adherens Junction Signaling”; “IL-8 Signaling” and “Acute Phase Response Signaling” (Fig 3 and S12 Table)

#### Identification of upstream regulators linked to mRNA expression changes in circulating white blood cells of cows with subclinical endometritis

The upstream analysis module of IPA identified 83 putative upstream regulators (p-value < 0.05). Three of them were associated with a mechanistic network with significant Z-score (Z-Score ≤ -2 or Z-Score ≥ +2, p-value of overlap < 0.05; S13 Table). The predicted top activated upstream genes included lipid A, a key component of lipopolysaccharides (Fig 4), as well cholesterol, pro-inflammatory cytokines (IL4, IL1), mature microRNA (miR-1900, miR-339-3p), a chromatin-associated enzyme poly(ADP-ribose) polymerase (PARP1) and a multifunctional nuclear protein implicated in DNA repair, NFkB inhibitor alpha complex (NFkB complex). In cows displaying SCE at 45–55 DPP, a chemical-kinase inhibitor (SB203580; Fig 4), Transcription factor G Protein Pathway Suppressor 2 (GPS2), 5'-prime-AMP-activated protein kinase (AMPK, a cellular energy sensor), Heme oxygenase 1 (HMOX1) that plays a critical role in inflammation and iron homeostasis were predicted to be top inhibited upstream genes.

#### Analysis of selected genes by quantitative real time PCR

**Fig 5:**
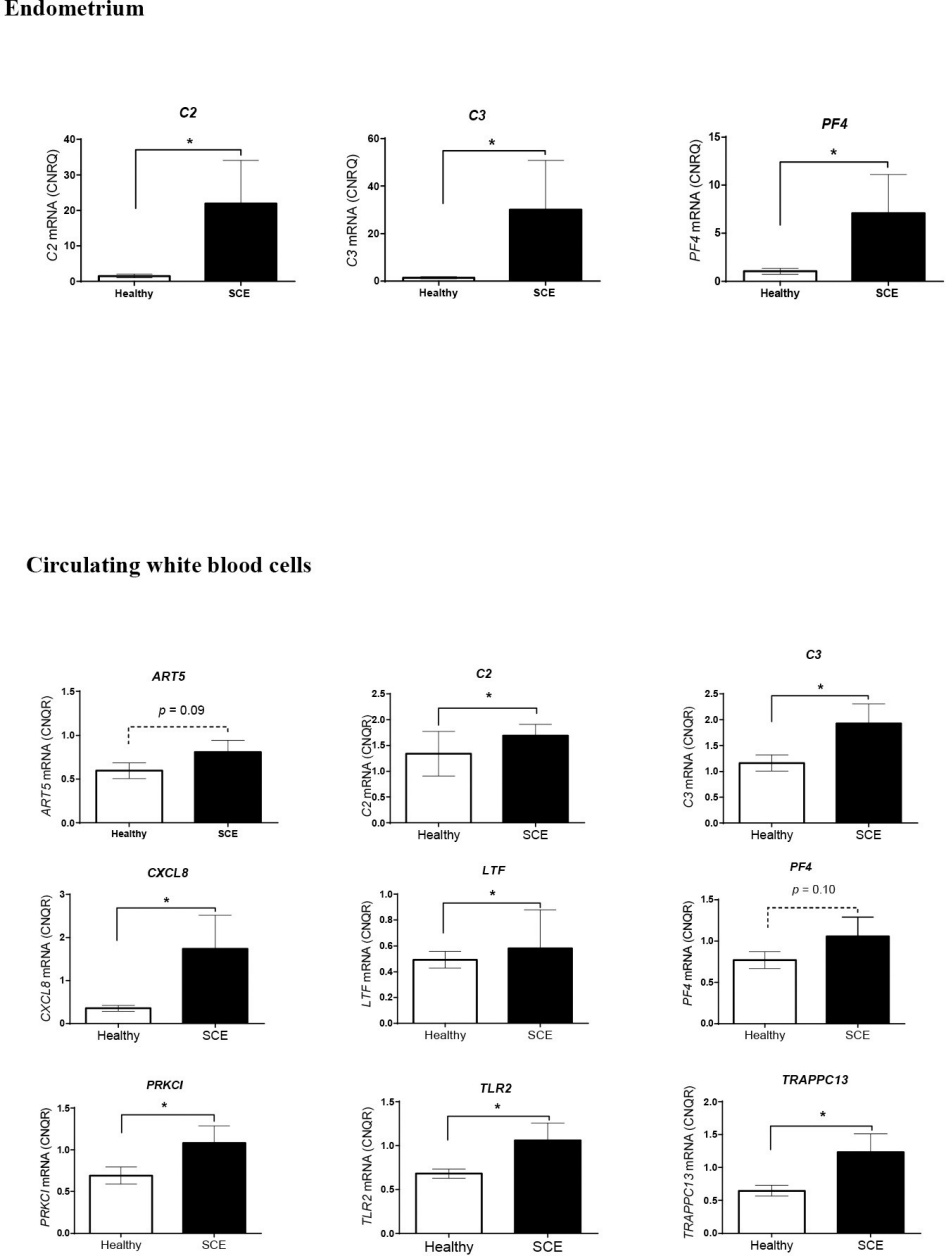
Gene expression in endometrium and circulating white blood cells of Holstein dairy cows at 45–55 days postpartum. In the **endometrium,** expression levels of *C2*, *C3* and *PF4*, gene transcripts were quantified by RT-qPCR analyses in endometrial biopsies collected from cows with subclinical endometritis (SCE, n = 3 in luteal phase) and from healthy cows (n = 10 including follicular phase, n = 5; luteal phase, n = 5) at 45–55 days postpartum (DPP). Relative gene expression was determined by normalization to the two most stable reference genes (*GAPDH* and *TPRG1L*). In **circulating white blood cells**, expression levels of *ART5*, *C2*, *C3*, *CXCL8*, *LTF*, *PF4*, *PRKCI*, *TLR2* and *TRAPPC13* gene transcripts were quantified by RT-qPCR analyses in cows with subclinical endometritis (SCE, n = 7, including follicular phase, n = 1; luteal phase, n = 4; undetermined estrous cycle, n = 2) compared with healthy cows (n = 10, including follicular phase, n = 5; luteal phase, n = 5) at 45–55 days postpartum (DPP). Relative gene expression was determined by normalization to the two most stable reference genes (*GAPDH* and *ACTB*). All expression data for gene transcripts of interest are expressed as mean calibrated normalized relative quantity (CNRQ) values in arbitrary units. The significance of differences in gene expression levels between groups of animals was determined by Kruskal Wallis, Dunn’s multiple comparisons test and by Mann Whitney U test at *p* < 0.05. Results are mean ± SEM.

To validate the microarray analyses in the endometrium, a selection of genes differentially expressed between SCE (n = 3) and healthy (n = 10) cows was quantified by real time PCR (RT-qPCR). Expression of *C2*, *C3* and *PF4*, transcripts was significantly higher (p ≤ 0.05) in the SCE cows than in the healthy females (Fig 5). In the “healthy” group, when the stage of estrous cycle was considered (follicular phase; n = 5 cows; luteal phase; n = 5 cows), RT-qPCR analysis showed that the physiological status (follicular phase or luteal phase) had a significant impact on the expression of several endometrial genes including *CAT*, *LTF*, *SAA3*, *SOD1*, *SOD2* and *TFF3* transcripts (S5 Fig). In the circulating WBC, no significant difference was detected between the LP and FP healthy cows that were merged in one category. Expression levels of *ART5*, *C2*, *C3*, *CXCL8*, *PF4*, *PRKCI*, *TLR2* and *TRAPP13* were higher in the SCE cows than in the healthy females (Fig 5).

#### Temporal regulation of genes in circulating white blood cells collected from SCE and healthy cows

To examine the impact of SCE on the temporal regulation of genes in circulating WBC, expression levels of nine candidate DEGs were determined by RT-qPCR during the postpartum period (1–4 DPP, 9–11 DPP and 45–55 DPP). Our results revealed that *ART5*, *C3*, *CXCL8*, *PF4*, *PRKCI*, *TLR2* and *TRAPPC13* were expressed in circulating white blood cells in a time-dependent manner during the postpartum period in healthy (n = 10) or SCE (n = 4) cows (Table 1 and S6 Fig). *TLR2* transcript level differed significantly between SCE and healthy cows at 1–4 DPP while *C3* was significantly higher in SCE cows at 9–11 DPP (S6 Fig).

**Table 1:** Temporal gene expression analyses (RT-qPCR) of selected candidate gene in circulating white blood cells of cows with subclinical endometritis during the post-partum period.

Gene symbol	Subclinical/Healthy			Healthy			Subclinical		
	DPP	Status	Status*DPP	1–4 vs. 9–11	1–4 vs. 45–55	9–11 vs. 45–55	1–4 vs. 9–11	1–4 vs. 45–55	9–11 vs. 45–55
*ART5*	0.002	0.155	-	-	0.055	0.026	-	0.059	0.038
*C2*	-	-	-	-	-	-	-	-	-
*C3*	0.001	0.073	0.006	-	0.194	0.193	0.001	0.002	-
*CXCL8*	-	-	0.012	-	0.003	0.093	-	-	-
*LTF*	-	-	-	-	-	-	-	-	-
*PF4*	-	-	0.030	-	0.036	0.026	-	-	-
*PRKC1*	-	-	-	-	-	0.159	-	-	-
*TLR2*	-	-	0.002	0.003	0.0001	-	0.191	-	-
*TRAPPC13*	-	-	0.026	-	0.017	0.030	-	-	-

Four subclinical females (follicular phase, n = 1, luteal phase, n = 1; undetermined estrous cycle, n = 2) and 10 healthy cows (follicular phase, n = 6; luteal phase n = 3; undetermined estrous cycle, n = 1) were used in this analysis. The significance of differences in gene expression levels between and within groups of animals was determined using Repeated Measures (RM) two-way ANOVA, matched values stacked into a subcolumn, Sidak’s multiple comparisons test at p-value < 0.05; “-”, not significant. Days: days post-partum (Status*days = interaction between the clinical status of cow and the day of post-partum period, 1–4; 9–11 and 45–55 days postpartum).

Additionally, the positions of selected candidate DEGs validated by RT-qPCR analyses in circulating WBC and endometrium were compared to the position on the UMD3.1 bovine genome assembly [33] of know quantitative trait loci (QTLs) deposited in the public database Animal QTLdb [34]. In total, 13 different genes were located in QTL regions related to female reproduction or infection traits (Table 2).

**Table 2:** Information on bovine Quantitative Trait Loci (QTL) related to reproduction traits overlapping with selected differentially expressed genes (DEGs).

Gene name	Gene description	BTA_QTL	QTL start	QTL end	Trait	QTL ID
*S100A8*	S100 calcium binding protein A8	3	16225278	18419617	Stillbirth (maternal)	126839
*CXCL8*	C-X-C motif chemokine ligand 8	6	87658297	92845663	Calving to conception interval	126853
*CXCL8*	C-X-C motif chemokine ligand 8	6	87658297	92845663	Interval to first estrus after calving	126854
*PF4*	Platelet factor 4	6	87658297	92845663	Calving to conception interval	126853
*PF4*	Platelet factor 4	6	87658297	92845663	Interval to first estrus after calving	126854
*TLR2*	Toll Like Receptor 2	17	3952978	3953018	Bovine tuberculosis susceptibility	106195
*LTF*	Lactotransferrin	22	53533293	53533333	Clinical mastitis	30827

## Discussion

The present study aimed to decipher the impact of SCE on circulating white blood cells and endometrium in high producing dairy cows. At Day 45–55 post-partum, using peripheral blood cells and endometrial biopsies sampled from control and SCE cows, transcriptome profiles were determined for circulating WBC and for endometrium of each cow. Our microarray results revealed that gene profiles were distinct between the endometrium and the circulating WBC. For each biological, compartment WBC, our statistical analyses also showed that several hundred genes were differentially expressed between SCE and healthy cows. These results suggest that SCE in high producing dairy cattle leads to alterations in gene expression of the systemic environment that are distinct from those determined in the endometrial tissue.

The observation that endometrium and WBC from SCE compared to healthy animals expressed high levels of mRNA encoding for mediators of inflammation including components of complement system and chemokines provided evidence that local inflammation is mediated not only by a combination of inflammatory cytokines produced by tissue-resident immune cells, but also by complex systemic processes. These results suggest that SCE is associated to a local and systemic activation of the complement system in dairy cattle at days 45–55 postpartum (DPP). Hence endometrial inflammation is associated with the complement system activation leading to the upregulation of *C2*, *C3* and *PF4* mRNA expression in SCE animals at 45–55 DPP. These results agree with recent study which reported that cows with SCE had higher mRNA expression of pro-inflammatory factors such chemokines (*CXCL1/2*, *CXCL3*, *CXCL5*, *CXCL8*,) and cytokines (*IL1A*, *IL1B*, *IL6*, *TNF*) in endometrium samples [35] and in cytobrush-epithelia cells on days 45–51 postpartum [36].

Several studies have demonstrated alterations in peripheral leukocyte populations and function in dairy cattle during the post-partum period [37,38,39]. Inflammatory cytokines and complement fragments have been reported to be mediators of leucocytes recruitment during the inflammation [40]. It could be suggested that peripheral mediators identified in our study are involved in neutrophil recruitment to the inflamed endometrium. More generally, further studies will be required to determine whether variations in peripheral leukocyte populations (cell type and cell number) account for variations in gene expression.

*C3* mRNA abundance was higher in WBC of SCE animals at early phase of postpartum period (9–11 DPP). Our results indicate that immune signatures may be predictive of SCE as early as 9–11 DPP in animals that are susceptible to develop SCE. C2 and C3 proteins mediate various inflammatory reactions and orchestrate both local inflammation and the development of adaptive immune responses [41,42]. The present study suggests that inflammation due to postpartum SCE is present in endometrium and peripheral blood cells in dairy cattle. This could be correlated with recent findings suggesting that changes in the number and composition of monocytes in the periphery predict the development of postpartum uterine disease in Holstein cows [43]. We found additional genes involved in innate immunity such *TLR2* and *CXCL8* were expressed at higher levels in circulating WBC from cows exhibiting postpartum SCE. Ours results are in keeping with published data reporting a similar increase of *CXCL8* mRNA expression in peripheral blood leucocytes of SCE cows 45–55 DPP [20] and *TLR2* mRNA in cows with mastitis [44]. *TLR2* is a cell-surface receptor that responds to microbial membrane components [45]. Based on the present study, we suggest that the abundance of *C2*, *C3*, *CXCL8*, *PF4* and *TLR2* mRNA expression in circulating white blood cells might represent useful markers for SCE in postpartum animals. Interestingly these result revealed a potential link between the activation of the complement system and SCE. Further studies are needed to explore how dysregulation of the complement system may be been implicated in the development of SCE in dairy cattle.

Interestingly, Muller et al. [46] identified several genomic regions associated with calving and fertility traits in Holstein. The location of three of selected candidate DEGs (*CXCL8*, *PF4* and *S100A8*) overlaps with three of these quantitative trait loci (QTL) regions. The *CXCL8* and *PF4* are located within regions associated with calving-to-conception interval or calving-to-first-estrous interval, while *S100A8* is within a stillbirth QTL region. A role of PF4 as a major player in the initiation and development of inflammatory diseases has been shown [47,48,49]. Others studies suggested that serum PF4 plays an important role as a potential biomarker of human chronic diseases, in particular inflammatory bowel disease [50]. Null mutations in murine *S100A8* show impaired early embryo development [51,52]. Mouse knock-out of the *C3* gene revealed that C3 plays an important role in early pregnancy [53]. Our study has not established whether the alteration of the expression of these genes may contribute to the negative impact on reproductive performance of SCE cattle, but this idea may be suggested. In human, it has been reported a potential linkage of severe preeclampsia to the most central complement gene, *C3* [54]. A broader genetic approach to identify SCE-related genes awaits further investigation [55,56].

Our data showed that circulating WBC collected from SCE cows expressed higher levels of *TRAPPC13* and *PRKCI* mRNA. *TRAPPC13* is a member of a multi-subunit TRAPP complexes (TRAPP; also known as trafficking protein particle), which are key players in the regulation of endoplasmic reticulum-to-Golgi transport and autophagy in yeast and mammals [57,58]. Studies have shown that TRAPPC8 is required for cell entry of the human papilloma virus [59]. *In vitro* study reported that the loss of mammalian TRAPPC13 in cells protects against Golgi-disrupting agents [60]. However, to our knowledge, no study has reported a potential molecular or cellular function of *TRAPPC13* in peripheral blood leucocytes of cattle. *PRKCI* is a member of PKC family which consists of multiple serine/threonine kinases regulating diverse cellular functions such cell proliferation, differentiation, and carcinogenesis and apoptosis [61]. Recent study provided evidence that *PRKCI* mRNA is highly expressed in human squamous cell carcinomas of lung [62]. Based on the cellular functions of *PRKCI*, our data might suggest that in diseased animals, SCE promoted apoptosis and compromises cell repair in circulating immune cells.

Most importantly the present study underlines a time-dependent expression pattern of candidate genes encoding components of complement system, chemokines, Toll-like receptors and factors of cell communication during the postpartum period in healthy cows compared with SCE animals. At 1–4 DPP, WBC from healthy cows expressed higher levels of *ART5*, *CXCL8*, *PF4*, *PRKCI*, *TRAPPC13* and *TLR2* than in SCE animals, and then mRNA levels declined at 45–55 DPP. Our results were supported by findings reporting lower levels of *IL-1beta* expression in peripheral blood mononuclear cell of cattle with acute puerperal metritis, which might indicate impaired inflammatory responses and may contribute to the development of metritis in disease cows [21]. Galvao *et al*. [63] also observed that bovine monocytes isolated from diseased cows and stimulated with bacteria *in vitro* exhibited lower mRNA expression of pro-inflammatory cytokines than healthy cows from calving to 14 DPP. However, in contrast to our observations, the authors observe no significant difference for *CXCL8* expression between groups. Furthermore, *C3* mRNA level in circulating WBC from healthy cows was low at the early phase and increased at the late postpartum phase. In this context the dynamic regulation of inflammation-related gene transcripts may indicate an earlier activation of immune system in healthy animals, which could prevent the development of SCE. We suggest that SCE may compromise early sufficient and effective activation of immune system, leading to the development of uterine disease. It has been reported that inflammatory factors are expressed in bovine endometrium in a time-related manner during the postpartum period [35,36]. Our results indicate that quantification of *CXCL8*, *PF4*, *TRAPPC13* and *TLR2* mRNA in peripheral leucocytes in the first week of postpartum period might provide useful predictive indicators of healthy and diseased animals. Our approach revealed that gene analyses in circulating WBC obtained at 9–11 DPP represent an additional source of information about the ability of animals to develop SCE. Further studies are necessary to clarify the link between the dynamic regulation of these genes and health status in cows during the postpartum period, relatively to fertility in healthy endometrium.

The present study indicates that endometrial mRNA expression of candidate genes was influenced by the stage of estrous cycle in cows during the postpartum period. In SCE endometrium, an increased expression of genes encoding constituents of mucus barriers such *TFF3* and genes encoding acute phase protein such as *SAA3*, was noticeable. In addition, our findings identified that “estrogen response late pathways signaling” as a major biological pathway perturbed in SCE at 45–55 DPP. Our study also demonstrates that antioxidant mechanisms operate within endometrium throughout the estrous cycle in healthy animals as previously demonstrated in sheep endometrium [64]. This finding might be related to the important role played by antioxidant enzymes in the regulation of uterine receptivity and the contribution of the endometrium to fertility [65].

## Conclusion

In summary our data indicate that mRNA expression alterations are associated with SCE in both circulating white blood cells and the endometrium of dairy cattle. Interestingly the peripheral and local responses to SCE are distinct in term of biological pathways and genes that are affected by the disease. In the endometrium, there was an increased expression of factors related to tissue remodeling, acute phase response, and LPS signaling. In circulating white blood cells, the main activated biological functions were linked to complement system, role of pattern recognition receptors, and movement of immune cells. The complement and the immune systems seem both to be modulated locally and systemically during SCE. During post-partum, expression of immune genes including *C3*, *CXCL8*, *LTF*, *TLR2* and *TRAPPC13* in circulating white blood cells represent potential peripheral indicators of health status that deserve to be tested at a broader scale.

## Supporting information

S1 Fig Peripheral blood leucocytes composition. (**A**) Principal Component Analysis using the number of leucocytes, polymorphonuclear cells (PMN), lymphoid cells and monocytes determined in peripheral whole blood collected from dairy Holstein cows with subclinical endometritis (SCE, n = 8) and in healthy Holstein dairy cows (n = 14) at 45–55 days postpartum. (**B**) Number of leucocytes, PMN, lymphoid cells and monocytes determined in peripheral whole blood collected from subclinical endometritis (SCE, n = 8) and healthy cows (n = 14) at 45–55 days postpartum independently of the estrous cycle). (**C**) Number of leucocytes, PMN, lymphocytes and monocytes determined in peripheral whole blood when estrous cycle is considered. SCE cows, n = 5 with follicular phase, n = 1; luteal phase, n = 4; healthy cows, n = 10 with follicular phase, n = 6; luteal phase, n = 4). The significance of differences in the number of cells between groups of animals was determined using Man-Whitney, t-test. Data are mean values. * = P < 0.05; ** = P < 0.01.
(TIF)

S2 Fig Ingenuity pathways molecule function networks in endometrium. Selected networks (based on the most important focus molecules) contain up-regulated and down-regulated differentially expressed genes (DEG) identified in endometrium of cows with subclinical endometritis (SCE, n = 4) compared with healthy (n = 4) cows at 45–55 days postpartum (DPP). Top functions in the selected networks are linked to (**A**) “Amino Acid Metabolism, Small Molecule Biochemistry, Lipid Metabolism”, with (**B**) “Cell-To-Cell Signaling and Interaction, “Cellular Movement, Hematological System Development and Function” and with (**C**) “Digestive System Development and Function, Organismal Functions, Lipid Metabolism”. Network displays nodes (genes/gene products) and edges (the biological relationship between nodes). Red and green shaded nodes represent up- and down-regulated gene expression, respectively. The color intensity of the nodes indicates the fold change increase (red) or decrease (green) associated with a particular gene. Solid line indicates a direct interaction between nodes (genes/gene products) and a dashed line indicates an indirect relationship between nodes. White symbols indicate neighboring genes that are functionally associated, but not included in the differentially expressed gene list. The shape of the node is indicative of its function (legend available online www.ingenuity.com).
(TIF)

S3 Fig Disease and biological functions predicted by Ingenuity Pathway Analysis software (IPA) to be increased or decreased in endometrium and circulating blood cells of subclinical dairy Holstein cows at 45–55 days postpartum. The disease and biological functions those were over-represented in endometrium biopsies and in circulating white blood cells (WBC) of cows with subclinical endometritis (SCE, n = 4) compared with healthy (n = 4) cows at 45–55 days postpartum (DPP). Green and red arrows indicated the disease and biological functions that were increased or decreased in endometrium biopsies of SCE cows compared with healthy cows respectively. The predicted activation state was determined by Z-Score value which is a statistical measure of the match between expected relationship direction and observed gene expression. Z-score ≤ **-**2 or Z-score ≥ 2 is considered significant. Ps were insignificant (p < 0.05) for random datasets calculated using Fisher’s exact test.
(TIF)

S4 Fig Ingenuity pathways molecule function networks in circulating white blood cells. Selected networks (based on the most important focus molecules) contain up-regulated and down-regulated differentially expressed genes (DEGs) identified in circulating white blood cells of cows with subclinical endometritis (SCE, n = 4) compared with healthy (n = 4) cows at 45–55 days postpartum (DPP). The most important functions in the selected networks are linked to (**A**) “Endocrine System Development and Function, Lipid Metabolism, Small Molecule Biochemistry”, (**B**) “Humoral Immune Response, Protein Synthesis, Hematological System Development and Function”, (**C**) “Cell Cycle, Reproductive System Development and Function, Cardiovascular Disease”. For more explications see legend in Supplemental S2 Fig.
(TIF)

S5 Fig Gene expression in endometrium and in circulating white blood cells of Holstein dairy cows at 45–55 days postpartum. (*Top panel*): Expression levels of *CAT*, *LTF*, *SAA3*, *SOD1*, *SOD2* and *TFF3*, gene transcripts were quantified by RT-qPCR analyses in endometrial biopsies collected from 3 cows with subclinical endometritis (SCE) and from 10 healthy cows that were subdivided in “follicular phase” group (n = 5) and “luteal phase” group (n = 5 at 45–55 days postpartum (DPP). (*Bottom panel*): Expression levels of *ART5*, *C2*, *C3*, *CXCL8*, *LTF*, *PF4*, *PRKCI*, *TLR2* and *TRAPPC13* gene transcripts were quantified by RT-qPCR analyses in circulating white blood cells collected from cows with subclinical endometritis (SCE, n = 7, follicular phase, n = 1, luteal phase, n = 4, undetermined estrous cycle, n = 2) and from 10 healthy cows (follicular phase, n = 5; luteal phase n = 5) at 45–55 days postpartum (DPP). Relative gene expression was determined by normalization to the two most stable reference genes (*GAPDH* and *TPRG1L*).
(TIF)

S6 Fig Temporal changes of gene expression in circulating white blood cells of Holstein dairy cows during the postpartum period. Expression levels of *ART5*, *C2*, *C3*, *CXCL8*, *LTF*, *PF4*, *PRKCI*, *TLR2* and *TRAPPC13* gene transcripts were quantified by RT-qPCR in 4 cows with subclinical endometritis (SCE, follicular phase, n = 1, luteal phase, n = 1; undetermined estrous cycle, n = 2) and 10 healthy cows (follicular phase, n = 6; luteal phase, n = 3; undetermined estrous cycle, n = 1) at 1–4, 9–11 and 45–55 days postpartum (DPP). Relative gene expression was determined by normalization to the two most stable reference genes (*GAPDH* and *ACTB*). All expression data for gene transcripts of interest are expressed as mean calibrated normalized relative quantity (CNRQ) values in arbitrary units. The significance of differences in gene expression levels between groups of animals was determined by Mann-Whitney U test at *p* < 0.05. Results are mean ± SEM.
(TIF)

S1 Table Classification of the animals used in this study according to health status and estrous cycle. “Undetermined”: when the stage of estrous cycle could not be obtained.
(DOCX)

S2 Table Primer sequences used for real time PCR quantification of transcript levels of housekeeping genes *(GAPDH*, *ACTB* and *TPRG1L*) and a selection of differentially expressed genes (DEGs). (XLSX)

S3 Table Differentially expressed genes (DEGs) identified in endometrium Holstein dairy cows (subclinical endometritis versus healthy; n = 4/condition) at 45–55 days postpartum. (XLSX)

S4 Table Differentially expressed genes (DEGs) identified in circulating white blood cells of Holstein dairy cows (subclinical endometritis versus healthy; n = 4/condition) at 45–55 days postpartum. (XLSX)

S5 Table Networks associated with differentially expressed genes in endometrium of Holstein dairy cows (subclinical endometritis versus healthy; n = 4/condition) at 45–55 days postpartum. (XLSX)

S6 Table Diseases and biological functions associated with differentially expressed genes in endometrium of Holstein dairy cows (subclinical endometritis versus healthy; n = 4/condition) at 45–55 days postpartum. (XLSX)

S7 Table Disease and Biological functions analysis of differentially expressed genes in subclinical compared with healthy dairy Holstein cows at 45–55 days postpartum (Ingenuity pathway analysis-IPA). Top 15 disease and biological functions (numbers of focus molecules and p < 0.05) are shown in table. The disease and biological functions those were over-represented in endometrium biopsies and in circulating white blood cells mRNA at 45–55 days post-partum (DPP) in cows with subclinical endometritis (SCE) compared with healthy cows. Ps were insignificant (p < 0.05) for random datasets calculated using Fisher’s exact test. The list of genes under each category is provided in S3 and S4 Tables.
(DOCX)

S8 Table Canonical pathways associated with differentially expressed genes in endometrium of Holstein dairy cows (subclinical endometritis versus healthy; n = 4/condition) at 45–55 days postpartum. (XLSX)

S9 Table Upstream regulators predicted to account for differences in gene expression observed in endometrium of Holstein dairy cows (subclinical endometritis versus healthy; n = 4/condition) at 45–55 days postpartum. (XLSX)

S10 Table Networks associated with differentially expressed genes in circulating white blood cells of Holstein dairy cows (subclinical endometritis versus healthy; n = 4/condition) at 45–55 days postpartum. (XLSX)

S11 Table Diseases and biological functions associated with differentially expressed genes in circulating white blood cells of Holstein dairy cows (subclinical endometritis versus healthy; n = 4/condition) at 45–55 days postpartum. (XLSX)

S12 Table Canonical pathways associated with differentially expressed genes in circulating white blood cells of Holstein dairy cows (subclinical endometritis versus healthy; n = 4/condition) at 45–55 days postpartum. (XLSX)

S13 Table Upstream regulators predicted to account for differences in gene expression observed in circulating white blood cells of Holstein dairy cows (subclinical endometritis versus healthy; n = 4) at 45–55 days postpartum. (XLSX)
