# Supplementary figures and images for: Subclinical endometritis in dairy cattle is associated with distinct mRNA expression patterns in blood and endometrium
Source: PLoS One. 2019 Aug 2;14(8):e0220244. doi: 10.1371/journal.pone.0220244 (PMC6677313; doi:10.1371/journal.pone.0220244)

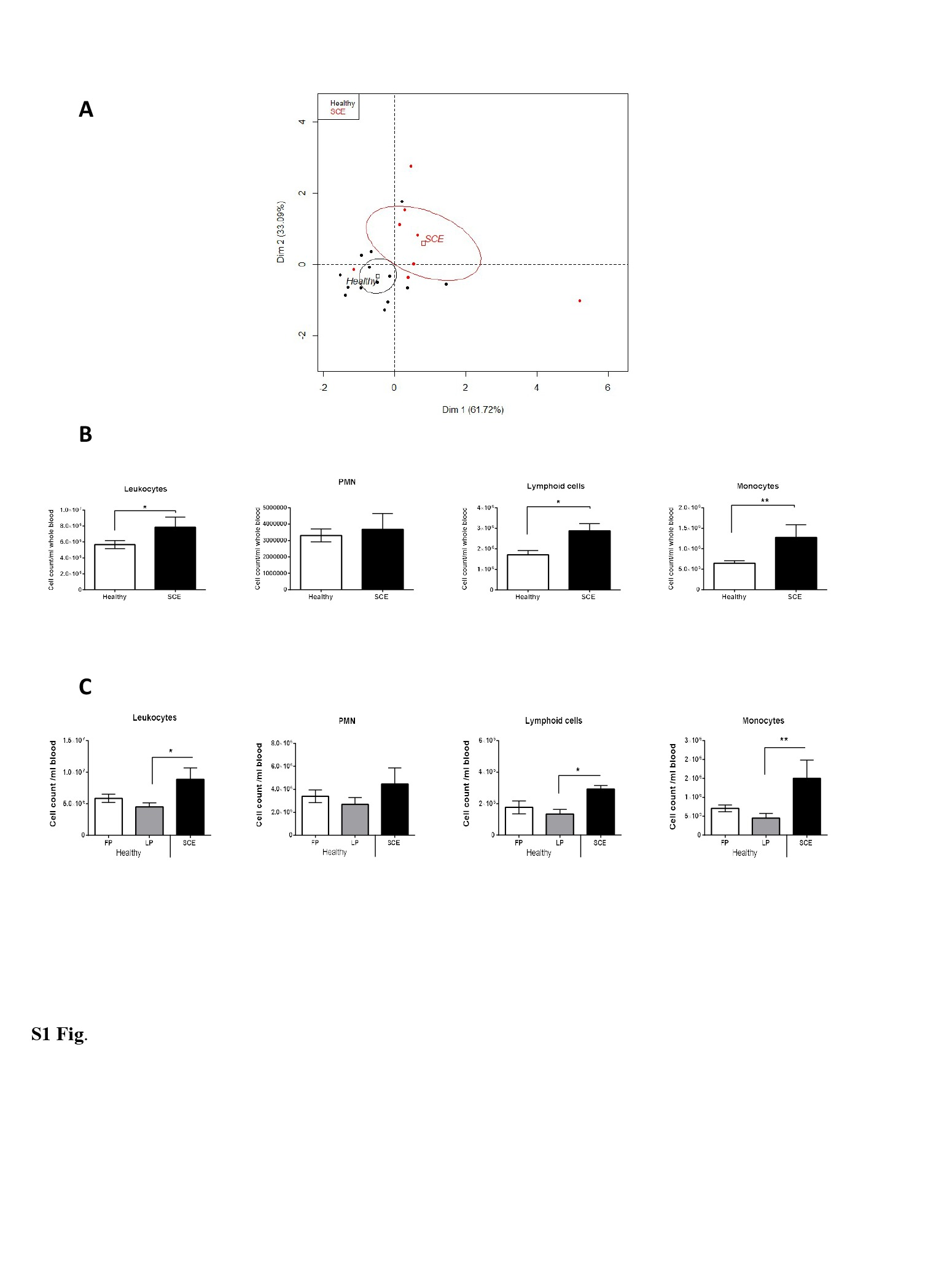

Supplement: S1 Fig — (A) Principal Component Analysis using the number of leucocytes, polymorphonuclear cells (PMN), lymphoid cells and monocytes determined in peripheral whole blood collected from dairy Holstein cows with subclinical endometritis (SCE, n = 8) and in healthy Holstein dairy cows (n = 14) at 45–55 days postpartum. (B) Number of leucocytes, PMN, lymphoid cells and monocytes determined in peripheral whole blood collected from subclinical endometritis (SCE, n = 8) and healthy cows (n = 14) at 45–55 days postpartum independently of the estrous cycle). (C) Number of leucocytes, PMN, lymphocytes and monocytes determined in peripheral whole blood when estrous cycle is considered. SCE cows, n = 5 with follicular phase, n = 1; luteal phase, n = 4; healthy cows, n = 10 with follicular phase, n = 6; luteal phase, n = 4). The significance of differences in the number of cells between groups of animals was determined using Man-Whitney, t-test. Data are mean values. * = P < 0.05; ** = P < 0.01. (TIF) [file pone.0220244.s001.tif]

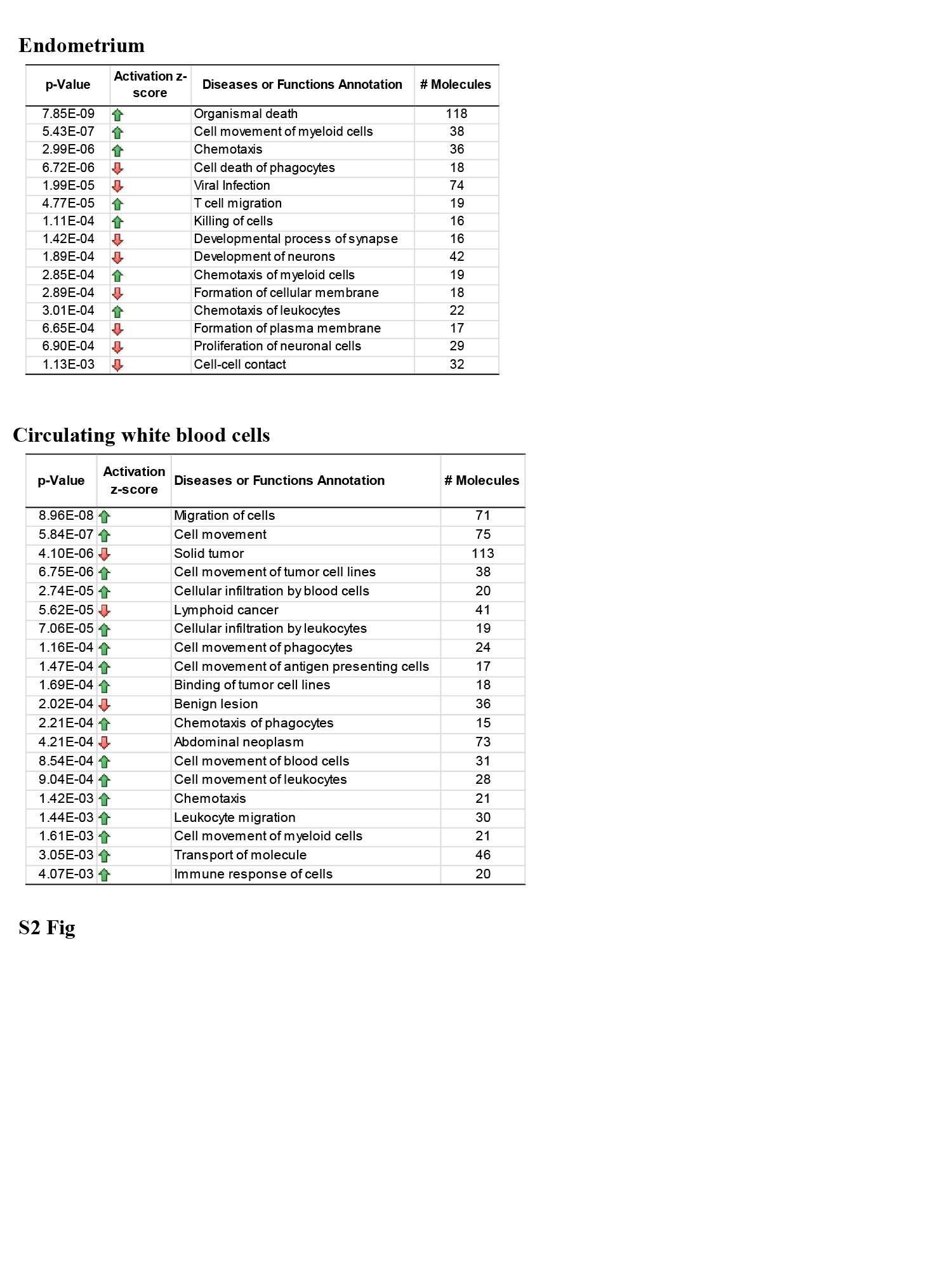

Supplement: S2 Fig — Selected networks (based on the most important focus molecules) contain up-regulated and down-regulated differentially expressed genes (DEG) identified in endometrium of cows with subclinical endometritis (SCE, n = 4) compared with healthy (n = 4) cows at 45–55 days postpartum (DPP). Top functions in the selected networks are linked to (A) “Amino Acid Metabolism, Small Molecule Biochemistry, Lipid Metabolism”, with (B) “Cell-To-Cell Signaling and Interaction, “Cellular Movement, Hematological System Development and Function” and with (C) “Digestive System Development and Function, Organismal Functions, Lipid Metabolism”. Network displays nodes (genes/gene products) and edges (the biological relationship between nodes). Red and green shaded nodes represent up- and down-regulated gene expression, respectively. The color intensity of the nodes indicates the fold change increase (red) or decrease (green) associated with a particular gene. Solid line indicates a direct interaction between nodes (genes/gene products) and a dashed line indicates an indirect relationship between nodes. White symbols indicate neighboring genes that are functionally associated, but not included in the differentially expressed gene list. The shape of the node is indicative of its function (legend available online www.ingenuity.com). (TIF) [file pone.0220244.s002.tif]

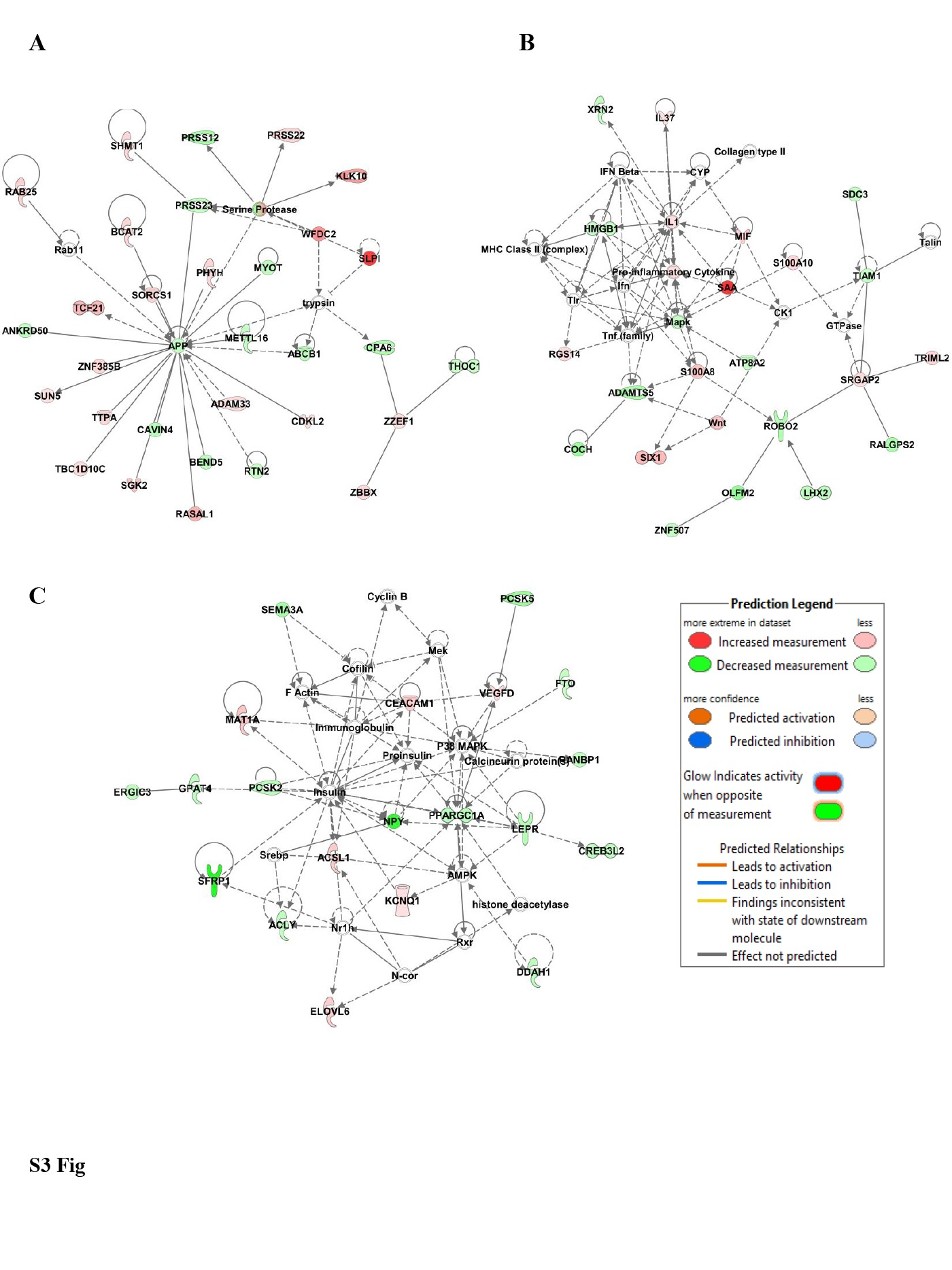

Supplement: S3 Fig — The disease and biological functions those were over-represented in endometrium biopsies and in circulating white blood cells (WBC) of cows with subclinical endometritis (SCE, n = 4) compared with healthy (n = 4) cows at 45–55 days postpartum (DPP). Green and red arrows indicated the disease and biological functions that were increased or decreased in endometrium biopsies of SCE cows compared with healthy cows respectively. The predicted activation state was determined by Z-Score value which is a statistical measure of the match between expected relationship direction and observed gene expression. Z-score ≤ -2 or Z-score ≥ 2 is considered significant. Ps were insignificant (p < 0.05) for random datasets calculated using Fisher’s exact test. (TIF) [file pone.0220244.s003.tif]

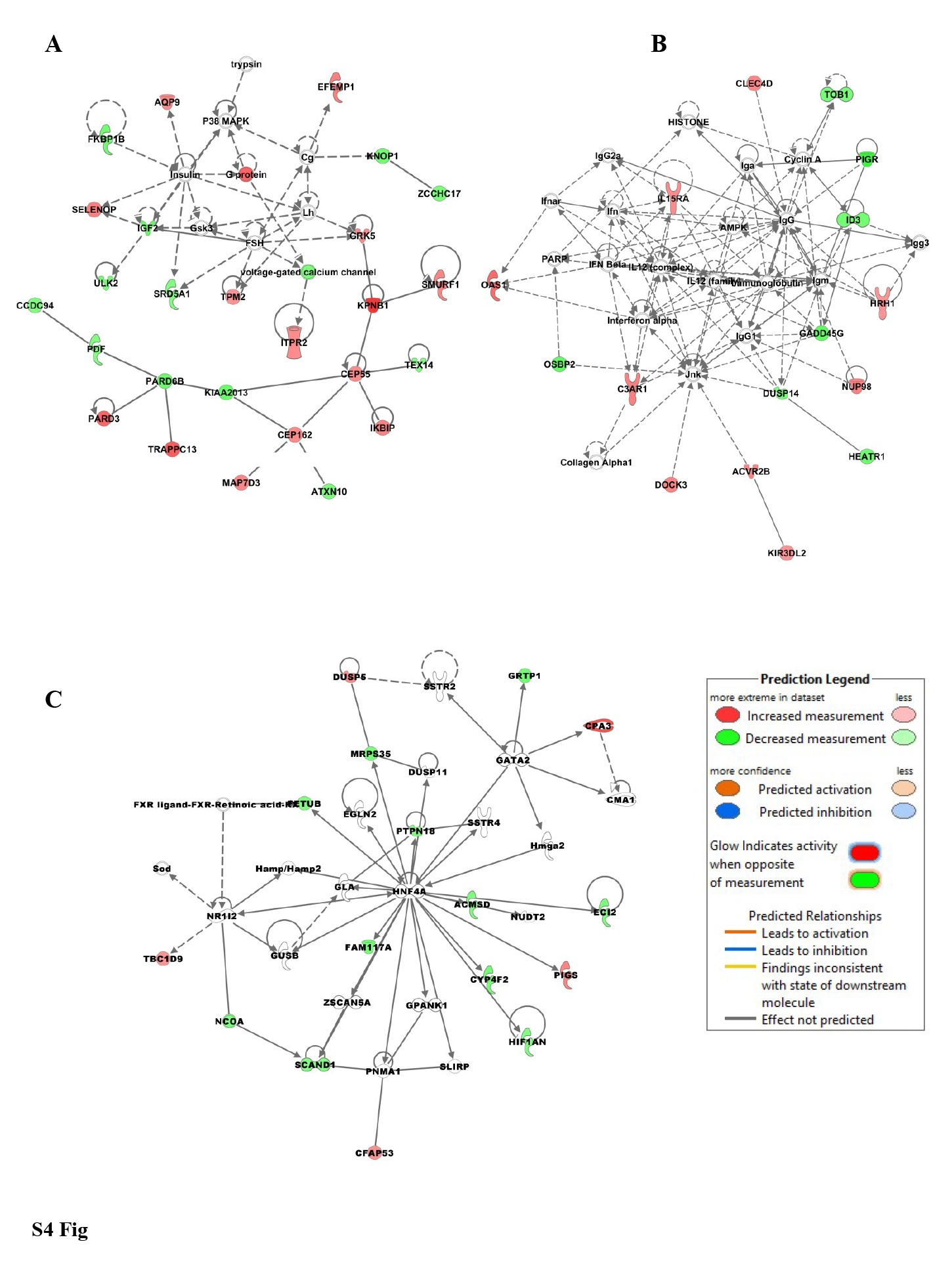

Supplement: S4 Fig — Selected networks (based on the most important focus molecules) contain up-regulated and down-regulated differentially expressed genes (DEGs) identified in circulating white blood cells of cows with subclinical endometritis (SCE, n = 4) compared with healthy (n = 4) cows at 45–55 days postpartum (DPP). The most important functions in the selected networks are linked to (A) “Endocrine System Development and Function, Lipid Metabolism, Small Molecule Biochemistry”, (B) “Humoral Immune Response, Protein Synthesis, Hematological System Development and Function”, (C) “Cell Cycle, Reproductive System Development and Function, Cardiovascular Disease”. For more explications see legend in Supplemental S2 Fig. (TIF) [file pone.0220244.s004.tif]

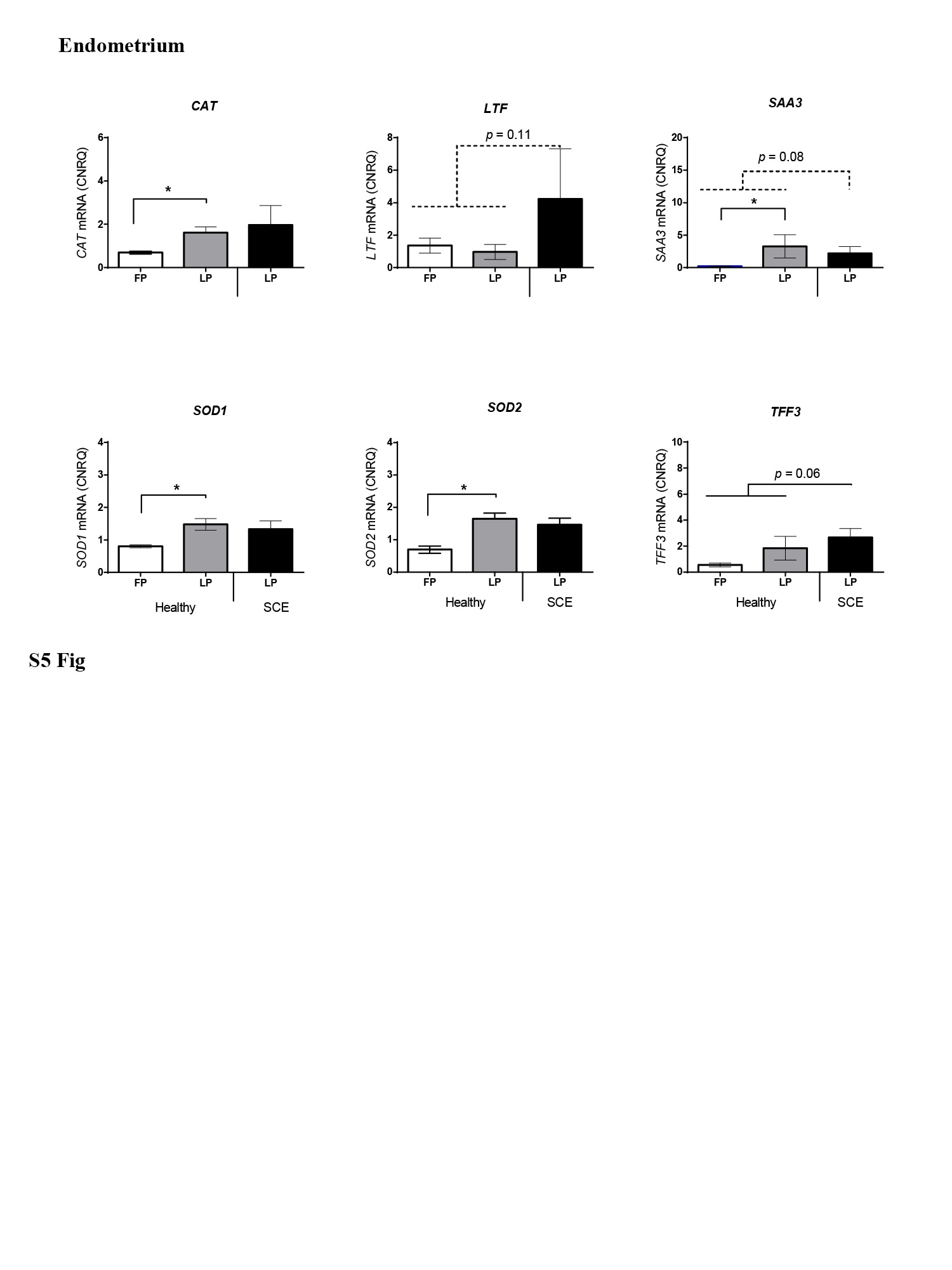

Supplement: S5 Fig — (Top panel): Expression levels of CAT, LTF, SAA3, SOD1, SOD2 and TFF3, gene transcripts were quantified by RT-qPCR analyses in endometrial biopsies collected from 3 cows with subclinical endometritis (SCE) and from 10 healthy cows that were subdivided in “follicular phase” group (n = 5) and “luteal phase” group (n = 5 at 45–55 days postpartum (DPP). (Bottom panel): Expression levels of ART5, C2, C3, CXCL8, LTF, PF4, PRKCI, TLR2 and TRAPPC13 gene transcripts were quantified by RT-qPCR analyses in circulating white blood cells collected from cows with subclinical endometritis (SCE, n = 7, follicular phase, n = 1, luteal phase, n = 4, undetermined estrous cycle, n = 2) and from 10 healthy cows (follicular phase, n = 5; luteal phase n = 5) at 45–55 days postpartum (DPP). Relative gene expression was determined by normalization to the two most stable reference genes (GAPDH and TPRG1L). (TIF) [file pone.0220244.s005.tif]

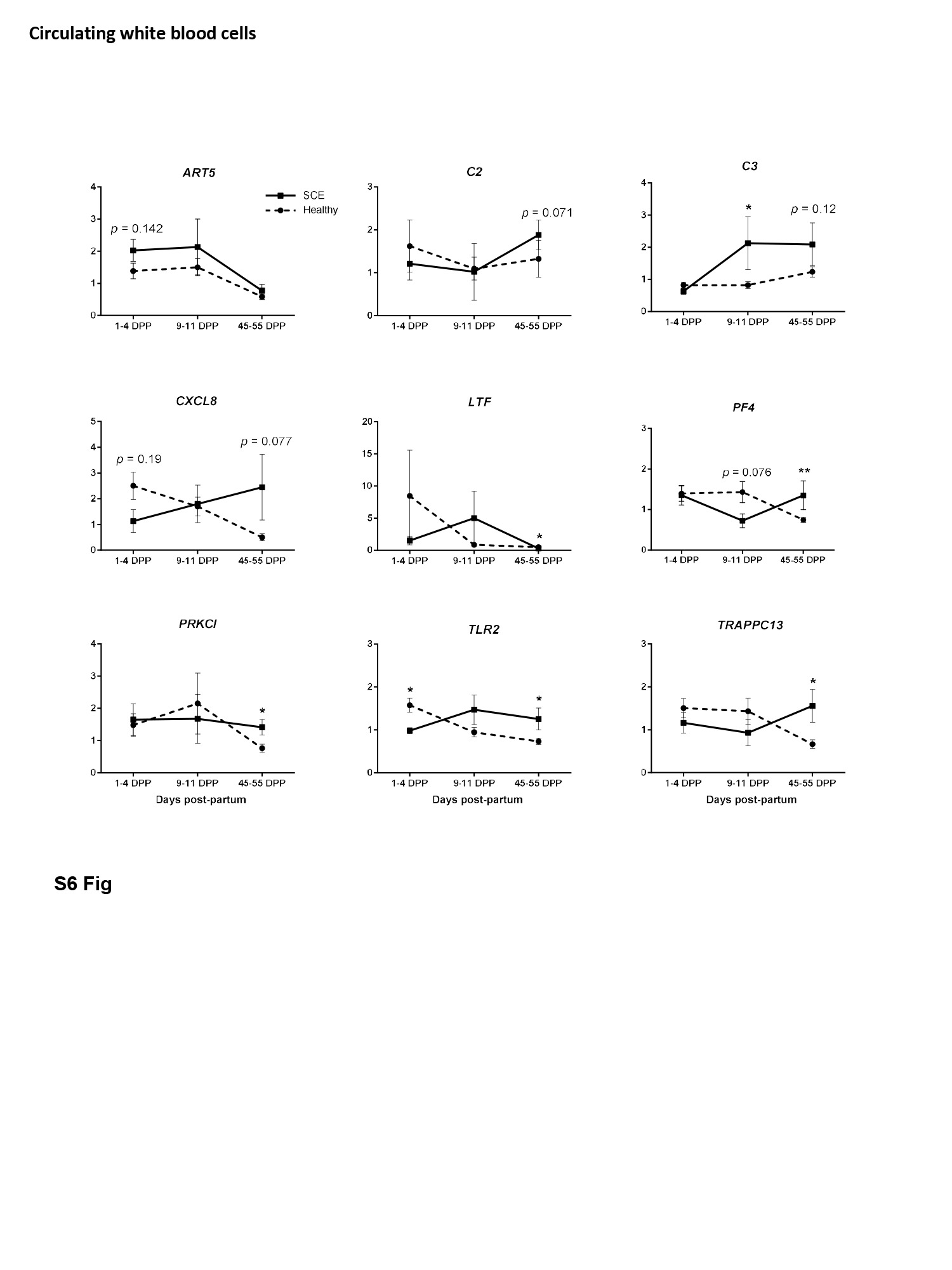

Supplement: S6 Fig — Expression levels of ART5, C2, C3, CXCL8, LTF, PF4, PRKCI, TLR2 and TRAPPC13 gene transcripts were quantified by RT-qPCR in 4 cows with subclinical endometritis (SCE, follicular phase, n = 1, luteal phase, n = 1; undetermined estrous cycle, n = 2) and 10 healthy cows (follicular phase, n = 6; luteal phase, n = 3; undetermined estrous cycle, n = 1) at 1–4, 9–11 and 45–55 days postpartum (DPP). Relative gene expression was determined by normalization to the two most stable reference genes (GAPDH and ACTB). All expression data for gene transcripts of interest are expressed as mean calibrated normalized relative quantity (CNRQ) values in arbitrary units. The significance of differences in gene expression levels between groups of animals was determined by Mann-Whitney U test at p < 0.05. Results are mean ± SEM. (TIF) [file pone.0220244.s006.tif]
