# Supplementary material for: Subclinical endometritis in dairy cattle is associated with distinct mRNA expression patterns in blood and endometrium
Source: PLoS One. 2019 Aug 2;14(8):e0220244. doi: 10.1371/journal.pone.0220244 (PMC6677313; doi:10.1371/journal.pone.0220244)
Supplement: S1 Table — “Undetermined”: when the stage of estrous cycle could not be obtained. (DOCX) [file pone.0220244.s007.docx]

| Cow phenotype | | | | Composition of peripheral blood cells | Microarrays (endometrium and WBC) | Total RNA available for RT-qPCR | | |
| --- | --- | --- | --- | --- | --- | --- | --- | --- |
| Health status | | estrous cycle | |  |  | 45-55 days post-partum | | Time course |
|  |  |  |  |  |  | WBC | endometrium | WBC |
| Healthy | 17 | Luteal phase | 6 | 4 | 2 | 5 | 5 | 3 |
|  |  | Follicular phase | 7 | 6 | 2 | 5 | 5 | 6 |
|  |  | undetermined | 4 | _ | _ | _ | _ | 1 |
| Subclinical endometritis (SCE) | 9 | Luteal phase | 4 | 4 | 3 | 4 | 3 | 1 |
|  |  | Follicular phase | 1 | 1 | 1 | 1 | _ | 1 |
|  |  | undetermined | 4 | _ | _ | 2 | _ | 2 |
