# Supplementary material for: Subclinical endometritis in dairy cattle is associated with distinct mRNA expression patterns in blood and endometrium
Source: PLoS One. 2019 Aug 2;14(8):e0220244. doi: 10.1371/journal.pone.0220244 (PMC6677313; doi:10.1371/journal.pone.0220244)
Supplement: S7 Table — Top 15 disease and biological functions (numbers of focus molecules and p < 0.05) are shown in table. The disease and biological functions those were over-represented in endometrium biopsies and in circulating white blood cells mRNA at 45–55 days post-partum (DPP) in cows with subclinical endometritis (SCE) compared with healthy cows. Ps were insignificant (p < 0.05) for random datasets calculated using Fisher’s exact test. The list of genes under each category is provided in S3 and S4 Tables. (DOCX) [file pone.0220244.s013.docx]

**Endometrium**

**Circulating white blood cells**
